# The pig X and Y Chromosomes: structure, sequence, and evolution

**DOI:** 10.1101/gr.188839.114

**Published:** 2016-01

**Authors:** Benjamin M. Skinner, Carole A. Sargent, Carol Churcher, Toby Hunt, Javier Herrero, Jane E. Loveland, Matt Dunn, Sandra Louzada, Beiyuan Fu, William Chow, James Gilbert, Siobhan Austin-Guest, Kathryn Beal, Denise Carvalho-Silva, William Cheng, Daria Gordon, Darren Grafham, Matt Hardy, Jo Harley, Heidi Hauser, Philip Howden, Kerstin Howe, Kim Lachani, Peter J.I. Ellis, Daniel Kelly, Giselle Kerry, James Kerwin, Bee Ling Ng, Glen Threadgold, Thomas Wileman, Jonathan M.D. Wood, Fengtang Yang, Jen Harrow, Nabeel A. Affara, Chris Tyler-Smith

**Affiliations:** 1 Department of Pathology, University of Cambridge, Cambridge CB2 1QP, United Kingdom;; 2 Wellcome Trust Sanger Institute, Hinxton, Cambridge CB10 1SA, United Kingdom;; 3 European Molecular Biology Laboratory, European Bioinformatics Institute, Hinxton, Cambridge CB10 1SD, United Kingdom;; 4 Bill Lyons Informatics Centre, UCL Cancer Institute, University College London, London WC1E 6BT, United Kingdom

## Abstract

We have generated an improved assembly and gene annotation of the pig X Chromosome, and a first draft assembly of the pig Y Chromosome, by sequencing BAC and fosmid clones from Duroc animals and incorporating information from optical mapping and fiber-FISH. The X Chromosome carries 1033 annotated genes, 690 of which are protein coding. Gene order closely matches that found in primates (including humans) and carnivores (including cats and dogs), which is inferred to be ancestral. Nevertheless, several protein-coding genes present on the human X Chromosome were absent from the pig, and 38 pig-specific X-chromosomal genes were annotated, 22 of which were olfactory receptors. The pig Y-specific Chromosome sequence generated here comprises 30 megabases (Mb). A 15-Mb subset of this sequence was assembled, revealing two clusters of male-specific low copy number genes, separated by an ampliconic region including the *HSFY* gene family, which together make up most of the short arm. Both clusters contain palindromes with high sequence identity, presumably maintained by gene conversion. Many of the ancestral X-related genes previously reported in at least one mammalian Y Chromosome are represented either as active genes or partial sequences. This sequencing project has allowed us to identify genes—both single copy and amplified—on the pig Y Chromosome, to compare the pig X and Y Chromosomes for homologous sequences, and thereby to reveal mechanisms underlying pig X and Y Chromosome evolution.

The therian (marsupial and eutherian) sex chromosomes evolved originally from a homologous pair of autosomes (Ohno 1967a) ∼170–180 million years ago (Livernois et al. 2012; Cortez et al. 2014) and have become extensively differentiated in terms of structure and sequence content. The gene content and organization of the emergent X Chromosome have been subject to strong conservation across different mammalian species with retention of much of the ancestral X (Ross et al. 2005; Bellott and Page 2009). In contrast, the acquisition of a dominant male sex-determining function and accumulation of male benefit genes (e.g., genes involved in regulating male germ cell differentiation) on the Y Chromosome has been accompanied by the genetic isolation of much of the Y through suppression of recombination with the emergent X, subsequent degeneration with loss of much of the ancestral Y gene content, and dosage compensation of genes on the X Chromosome to restore equivalence of gene expression between males and females (Graves 2010; Bachtrog 2013). Selection has also acted to retain a strictly X-Y homologous pseudoautosomal region (PAR) that permits X-Y pairing during meiosis and within which there is obligate recombination between the sex chromosomes. The gene and sequence content of the PAR varies between species, reflecting processes of expansion and pruning of the PAR in different mammalian lineages (Otto et al. 2011).

Comparisons of X Chromosome sequences from several mammalian species have confirmed strong conservation of gene sequence and order (Chinwalla et al. 2002; Sandstedt and Tucker 2004). Groenen et al. (2012) published the first assembly of the porcine X Chromosome as part of the initial description of the pig genome sequence, and again this demonstrated conservation of synteny across the chromosome. Nonetheless, sequence gaps and ambiguities remained within this first assembly, complicating genomic studies within pigs and comparative studies between mammals.

In contrast to the broadly conserved X Chromosomes, the hemizygous nature of the Y Chromosome and suppression of recombination, in combination with normal processes of genome evolution, have led to a gradual degeneration of the chromosome over time, chromosomal rearrangements, and colonization by sequences from the X Chromosome and autosomes. Newly introduced genes will either drift or degenerate, or selection may act on variants to fix new genetic functions on the Y, particularly where these confer a male benefit. The haploid state of the sex chromosomes in males has led to the accumulation of male gametogenesis genes on both X and Y Chromosomes (Vallender and Lahn 2004). A further consequence of the nonrecombining status of the male-specific region of the Y is the relaxation of restraint on sequence amplification, leading to generation of ampliconic regions containing amplified gene and sequence families (Bellott and Page 2009).

The highly repetitive nature of many regions of mammalian Y Chromosomes has impeded the generation of complete chromosome sequences; while there are tens of mammalian genomes sequenced, only a small fraction have a Y assembly. These few assemblies, and several partial sequence assemblies, have permitted the elucidation of chromosome topology and gene order in human (Skaletsky et al. 2003), chimpanzee (Hughes et al. 2010), rhesus macaque (Hughes et al. 2012), wallaby (Murtagh et al. 2012), mouse (Soh et al. 2014), marmoset, rat, and opossum (Bellott et al. 2014), cattle (Elsik et al. 2009), horse (Paria et al. 2011), and cat and dog (Li et al. 2013a). These works show divergence in gene content, order, structure, and sequence between Y Chromosomes from different mammalian species. However, few data are available on the porcine Y Chromosome sequence or gene order, and their relationship to the X, despite the recent sequencing project for the pig genome (Groenen et al. 2012), remains unclear. Much of our knowledge of Y gene order comes from Quilter et al. (2002), who combined radiation hybrid mapping data with physical mapping of BAC clones to generate an ordered gene framework.

The current work presents a second-generation, much improved assembly and gene annotation of the porcine X Chromosome based on the Duroc X Chromosome. We also present the Y Chromosome sequence derived predominantly from Duroc, with some Meishan, which has permitted a first-generation assessment of the Y Chromosome short-arm gene content and order, and analysis of how this compares to other mammalian Y Chromosomes, the evolutionary processes leading to the current Y organization, and the structural relationships between the porcine sex chromosomes.

## Results

### A second-generation porcine Chromosome X assembly

#### Sequence statistics

The Chromosome X assembly (http://vega.sanger.ac.uk/Sus_scrofa/Location/Chromosome?r=X-WTSI) comprises 129,927,919 bp of sequence in five contigs, with 13 gaps and an N50 length of 4,824,757 bp. Compared with the previous 10.2 build, many gaps have been filled and the order of sequences on the chromosome has been updated. Much of this improvement was informed by the use of optical mapping techniques, which helped resolve some of the more repetitive regions of the chromosome; an example can be seen in the short-arm clone CH242-202P13 (see Supplemental Methods Fig. OM7 for details on how the optical mapping approach was used here). Supplemental Figure S3 shows a dot-plot alignment of the 10.2 X with our X assembly, highlighting the regions of the chromosome for which the sequence order has been corrected.

The pseudoautosomal region in pig is of a similar size to the PAR in other closely related mammals (e.g., cattle) and has been discussed previously (Skinner et al. 2013). The precise location of the PAR boundary was recently confirmed to be within the gene *SHROOM2* (Das et al. 2013). A 33-kb region containing a lincRNA with homology to the PAR (X-WTSI: 1,840,693–1,874,130) can be found on Xq (X-WTSI: 114,853,327–114,886,764), and most likely arose via duplication and transposition from the PAR onto Xq.

#### Comparative X alignments

**Figure 1.**
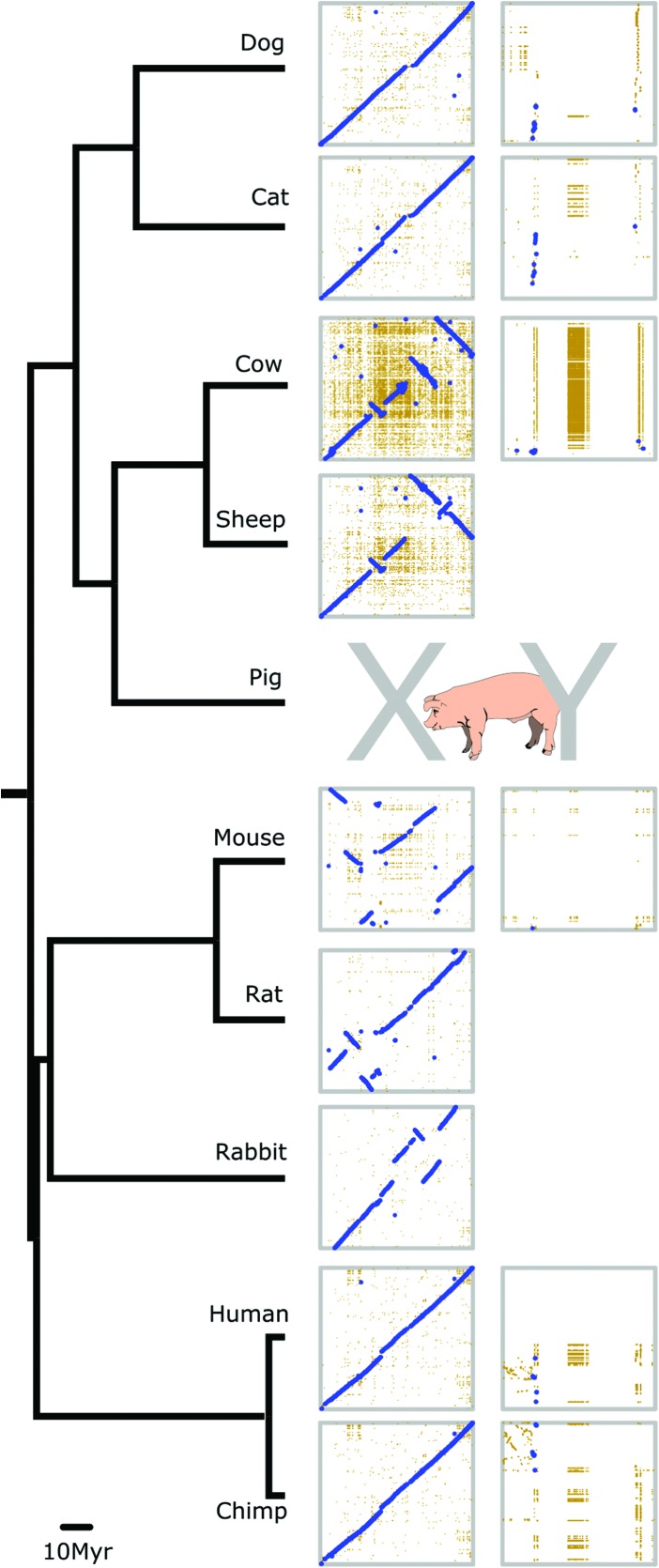
Comparative X and Y map. Sequenced X Chromosomes from nine mammals, plus available Y Chromosomes, aligned to our pig X and Y assemblies. In each dotplot, the pig chromosome is on the horizontal axis, and the subject chromosome is on the vertical axis. The cattle X sequence is plotted in reverse orientation. High-stringency alignments are shown in blue with less stringent alignments in yellow. Human, chimpanzee, cat, and dog retain the ancestral X arrangement. Sheep and cattle show a small number of rearrangements, while rodents and rabbit have a greater rate of chromosomal change. Chromosomes derived from shotgun assemblies are more prone to showing rearrangement and reflect the need for continuous assembly improvement. The Y alignments show highly variable organization, and different ancestral genes have amplified in different lineages (note that the sizes of the Y assemblies are not to scale here; see Supplemental Fig. S8 for larger versions).

We aligned the X (and available Y) Chromosome sequences of nine mammalian species (Fig. 1). The previously documented high level of conservation of synteny is more apparent with the new pig X, as many of the reported breakpoints from cross-species comparisons to the build 10.2 X were due to errors that have been resolved. Li et al. (2013b) produced a genome assembly of a female Tibetan wild boar and reported regions of the genome with apparent inversions with respect to the Duroc assembly. We compared the X inversions with our new assembly and found that they lie outside the regions that have been resolved from the 10.2 X. That is, these remain as potential inversions between Duroc and Tibetan wild boar that require further investigation.

#### Gene content of the X

Chromosome X contains complex duplicated gene families such as olfactory receptors (OR) along with pseudogenes, which are hard to discriminate using only automatic pipelines. Since the reference assembly was high quality, the sequence underwent manual annotation to allow resolution of these gene families. Table 1 shows the updated annotation compared to the manual annotation available on the 10.2 X build and comparable statistics for the manual annotation of the Y Chromosome. The full gene annotation is provided in Supplemental Table S5 and is available through the Vega website. The majority (76%) of annotated loci in pig are shared with human. Many genes from the previous build that were disrupted by gaps have now been completed, and the number of long noncoding RNA loci has increased from 33 to 100.

**Table 1.**
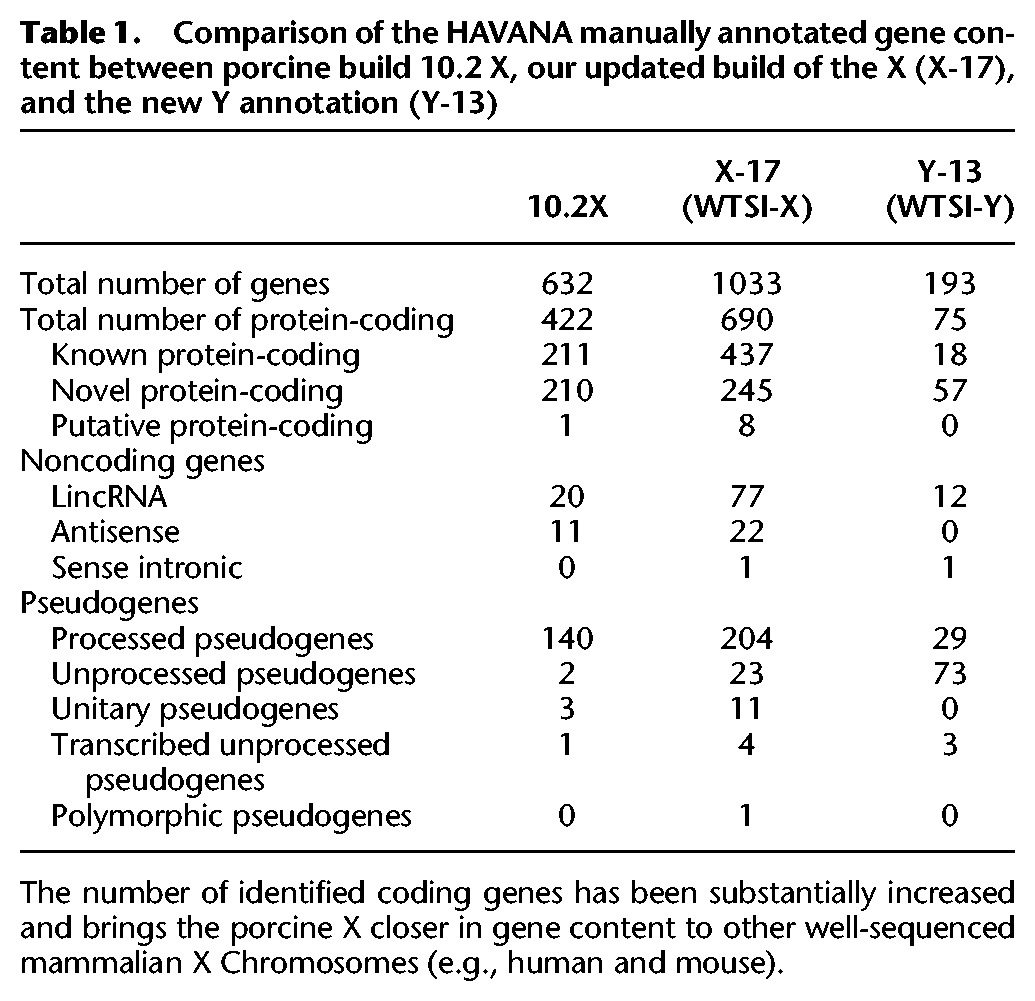
Comparison of the HAVANA manually annotated gene content between porcine build 10.2 X, our updated build of the X (X-17), and the new Y annotation (Y-13)

Some genes with updated annotation from build 10.2 stand out as being of particular biological interest. Comparing the human and pig X Chromosomes, 38 coding loci in pig are not found in human. Twenty-two of these coding genes and five novel pseudogenes are in olfactory gene clusters. Pigs are known to have a large olfactory receptor repertoire (Nguyen et al. 2012), and this adds to the reported collection. Supplemental Figure S4 shows the improved assembly and annotation around one of the olfactory region clusters on Xq. The region lies within an inversion in the 10.2 assembly, corrected here and matching the gene order on the human X. The full list of genes present on pig Chromosome X, but not on the human X, is provided in Supplemental Table S7.

### A first-generation porcine Chromosome Y sequence assembly

Successfully assembled repetitive portions of Y Chromosomes have been generated only for a limited number of species (see, for example, the human, mouse, or chimpanzee Y) (Skaletsky et al. 2003; Hughes et al. 2010; Soh et al. 2014). Given the highly repetitive nature of the long arm of the pig Y Chromosome, we targeted the short arm, which contains most, if not all, of the single-copy material. The details of sequenced clones are given in Supplemental Table S1; the total sequence produced, including finished and unfinished clones, without overlaps covers 29,953,871 bp, with 15,592,828 bp annotated and placed in the Vega assembly (http://vega.sanger.ac.uk/Sus_scrofa/Location/Chromosome?r=Y-WTSI). Here, we focus on the assembled and ordered contigs within that sequence.

### Organization of the porcine Y Chromosome

**Figure 2.**
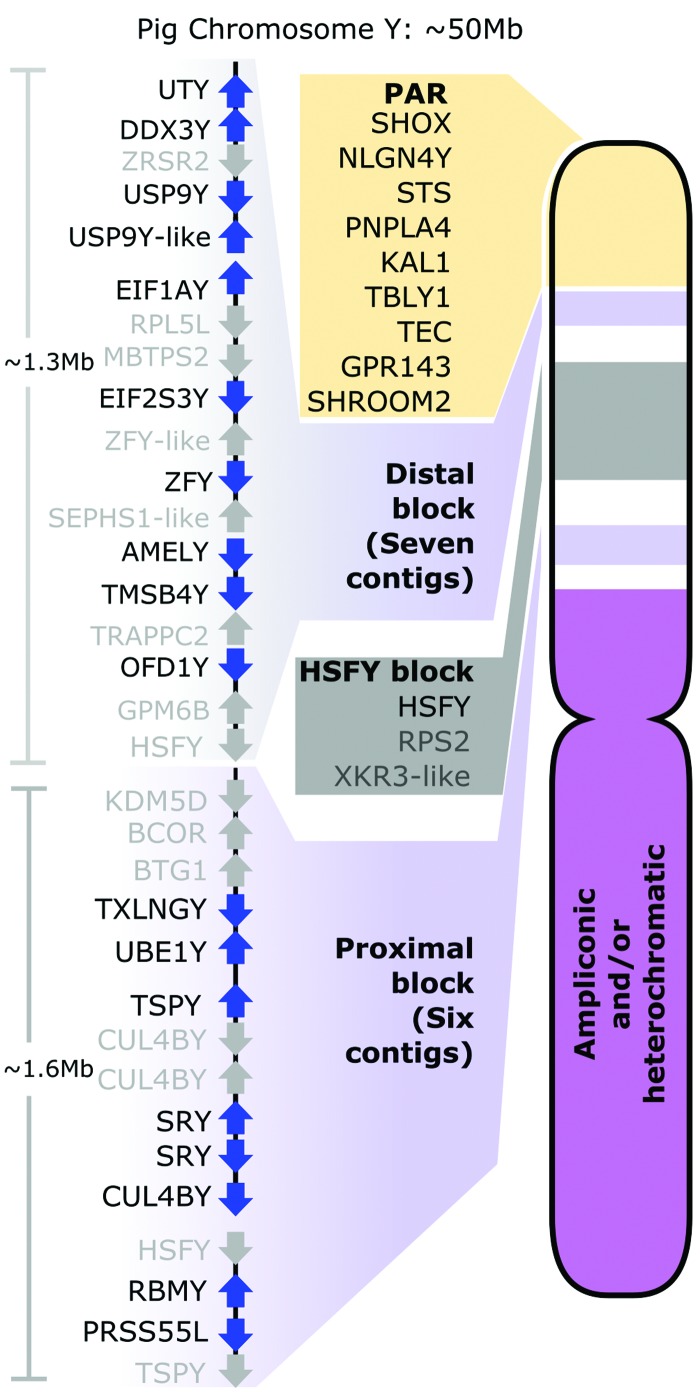
The organization of the pig Y Chromosome. All identified male-specific single-copy genes are on the short arm and split into two blocks by the ampliconic *HSFY* region. Genes (blue) and pseudogenes (gray) are shown within each block. The long and short arms toward the centromere appear to contain ampliconic or other repetitive sequences.

The genes on Chromosome Y are organized into two main blocks of low copy number sequences (Fig. 2). These blocks are separated by a region containing highly amplified sequences including ∼100 copies of the *HSFY* gene family (Skinner et al. 2015). Our final mapped sequence comprises seven contigs each in the distal and proximal blocks (Supplemental Fig. S5). Contigs that were assembled, but could not be assigned to the physical map, are included in a separate assembly (U_Y) in Vega. Two of these contigs appear to lie on Yp close to the centromere (Supplemental Figs. S6, S7).

Few low-copy loci have been identified on the Y long arm, which seems mostly composed of repetitive sequences (e.g., Quilter et al. 2002). Although we attempted to sequence one repetitive clone, it was not possible to assemble a framework physical or sequence map. The sequences we obtained belong to previously published male-specific pig repeat families (McGraw et al. 1988; Mileham et al. 1988; Thomsen et al. 1992; Pérez-Pérez and Barragán 1998). Metaphase FISH did, however, reveal a small low-copy region at the q terminus (Supplemental Fig. S13).

#### Gene-related content of the Y Chromosome

As with the X Chromosome, the Y-chromosomal sequence was run through the Otterlace/Zmap analysis pipeline, which performs homology searches, de novo sequence analysis, and gene predictions (Loveland et al. 2012). Repeat masking proved challenging due to the paucity of known pig-specific repeats. Manual annotation resolved this, as homology analysis is routinely run on-the-fly within the annotation tools, without repeat masking, to more accurately elucidate gene structures, especially using known Y-chromosomal genes from other species as targets and identifying those pig homologs present. Many of the ancestral X-related genes previously reported in other mammalian Y Chromosomes are represented either as active genes or partial sequences, some of the latter with supporting ESTs. There is also evidence for rearrangement of a number of Y-linked genes, which may have rendered them nonfunctional or modified their transcription products. An overview of Y gene loci is given in Table 2, and the full Y gene annotation table is provided as Supplemental Table S6. The contigs here show no evidence for novel pig genes, though these may yet be found in the ampliconic regions of the chromosome.

**Table 2.**
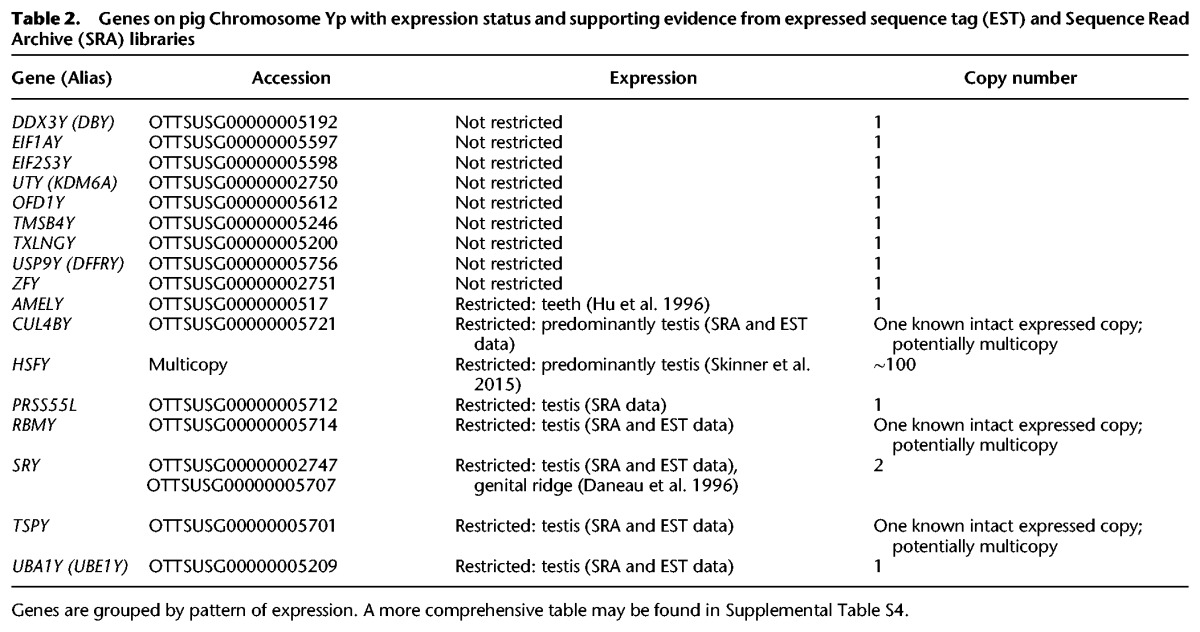
Genes on pig Chromosome Yp with expression status and supporting evidence from expressed sequence tag (EST) and Sequence Read Archive (SRA) libraries

#### Ampliconic gene sequences

Although our sequence contigs are limited to the low-copy regions of the chromosome, the FISH data show regions where gene sequence-containing clones are present in multiple copies. An example is the *CUL4BY* gene, which has a partial copy in fosmid WTSI_1061-13A5. Probes detecting this sequence bind multiple targets proximal to *SRY* (and likely proximal to *RBMY*), as well as additional sites toward the centromeric end of the Y short arm (Supplemental Fig. S13F). The sequence supports gene expression from a “full-length” version of the sequence centromeric to *SRY*, and RT-PCR shows expression in testis, kidney, and brain (Supplemental Fig. S2). A similar pattern of amplification exists for fosmid clones containing *TSPY*.

#### Regions of X-Y homology

**Figure 3.**
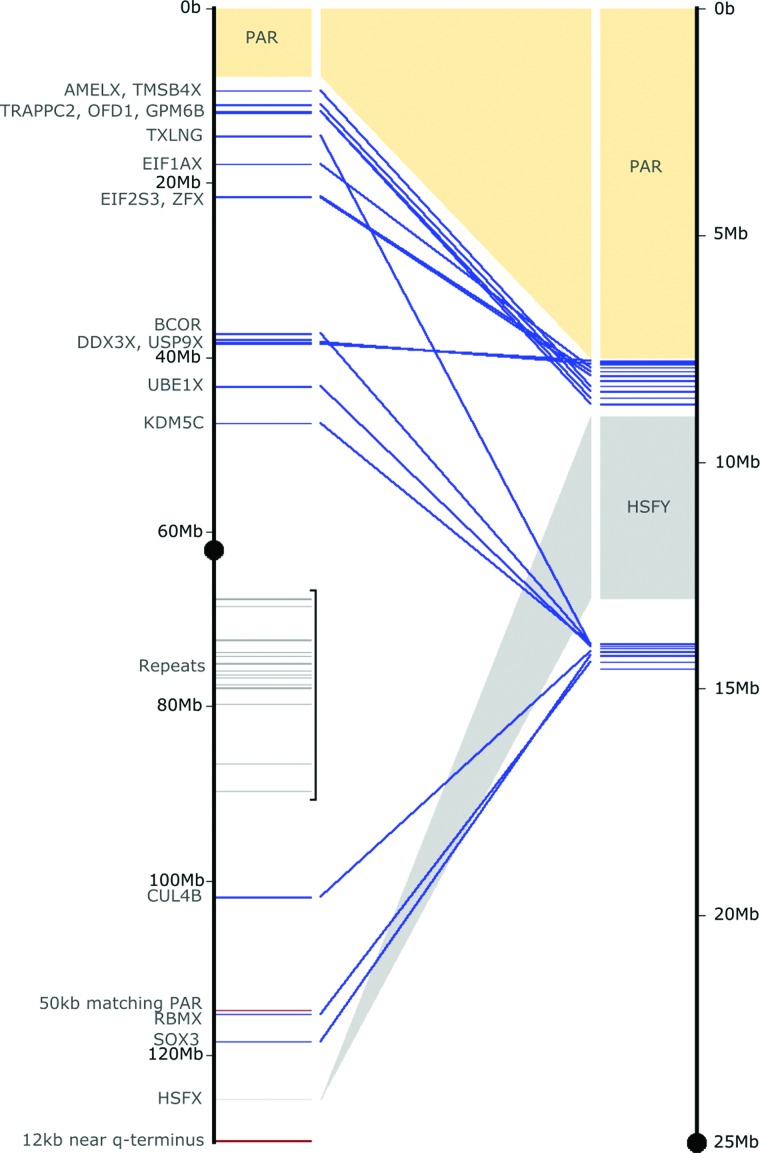
Homology between the X and Y. Outline of X-Y homology regions detected between the pig X and Yp sequences. Centromeres are black dots; the PAR is yellow. Lines for genes are blue, repetitive content is gray, and sequences as yet unplaced on the Y are red. The 50-kb region at X-114 Mb is an X-X transposition from the PAR. The amplified HSFY region is shown in dark gray. Regions of repetitive content correlate well with CGH patterns we found previously (Skinner et al. 2013).

We examined all sequenced Y clones for homology with the X. The overview X-Y comparison for the short arm of the Y is shown in Figure 3. Besides the X-Y homologous genes, a region of 90.25 kb on the distal block encompassing the genes (or gene fragments) *TRAPPC2-OFD1Y-GPM6B* has high sequence identity to the X, even in intronic and intergenic regions (Supplemental Fig. S1). *OFD1Y* is expressed highly in testis with lower expression in kidney and brain (Supplemental Fig. S2), but there is no evidence for expression of the Y copies of *TRAPPC2* or *GPM6B*. Previous work suggested that three X BAC clones around the olfactory receptor genes contain a sequence amplified on Yq; we did not find matches to these clones within our Y sequence data beyond repetitive elements; the potential for olfactory receptor-related sequences on the Y nevertheless remains.

### Evolution of the porcine Chromosome Y

#### Inverted and duplicated blocks of sequence

**Figure 4.**
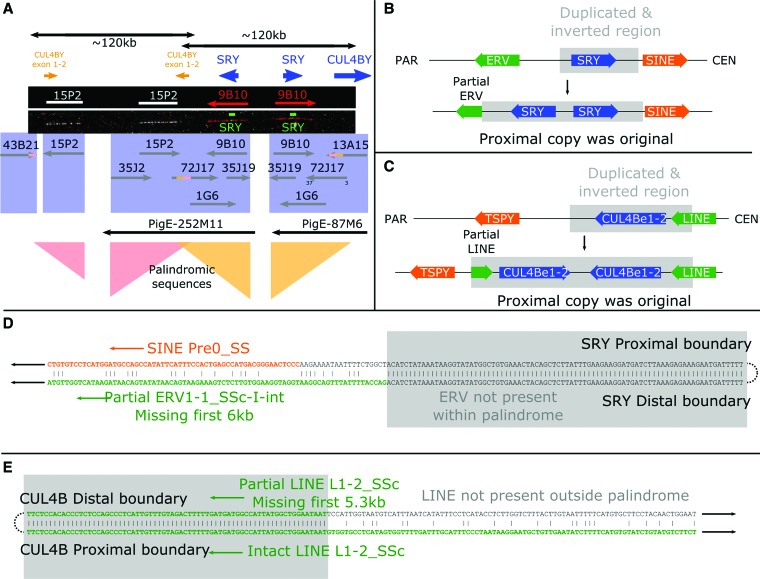
The pig *SRY* region. The Yp proximal block of genes contains two overlapping palindromes of ∼120 kb each. These surround the duplicated sequences *CUL4BY* exons 1–2 and *SRY*. (*A*) FISH results from Y fosmid clones and probes for the *SRY* gene are shown with the BAC and fosmid clone sequences found mapping to the region. The inversion boundaries are both identifiable; the *CUL4BY* inversion runs from the last 3 kb of 43B21 to within 72J17; the *SRY* inversion begins also within 72J17 and runs to 13A15. A schematic view is also shown of the regions surrounding the *SRY* (*B*) and the *CUL4B* duplications (*C*). The *SRY* duplication disrupts an ERV element, revealing the proximal copy to be ancestral. The *CUL4B* duplication copies part of a LINE element, again revealing the proximal copy to be ancestral. The sequence alignments across the inversion breakpoints are shown in more detail for *SRY* (*D*) and *CUL4B* (*E*). The order of events was therefore a duplication of *SRY*, including the first two exons of *CUL4B*, followed by duplication of the region around the *CUL4B* copy.

Inversion and duplication to form palindromes has occurred around both *SRY* and the *CUL4BY* fragments (Fig. 4). The *SRY* gene itself is present in two head-to-head copies. There are unlikely to be more than two *SRY* copies on the chromosome (qPCR) (Supplemental Fig. S10). The pattern of markers at the breakpoint regions reveals that the *SRY* duplication preceded the *CUL4B* duplication (see Fig. 4; Supplemental Fig. S9). Transposable elements at both inversion boundaries are annotated as specific to the *Sus* lineage, suggesting these are relatively recent duplications; two copies of *SRY* could also be present in closely related suid species. The arms of the palindrome have high levels of sequence identity; we found no difference in the *SRY* sequence from clones on one arm versus the other arm. Our sequence contigs do not cover the centers of the palindromes (∼20 kb is missing in each), so we do not know if the arms abut—it remains possible that there is a short stretch of unique sequence between them. Prior sequencing of the *SRY* gene has given no evidence for polymorphisms in the recovered *SRY* sequence from any individual, despite there being breed-specific differences—i.e., there are no heterozygous males identified (more than 300 *SRY* sequences are currently deposited in NCBI for *S. scrofa* alone). A further palindromic region lies at the proximal end of *USP9Y*, covering 56 kb, including the final exons 18–43 of the gene (Supplemental Fig. S11). Compared to *SRY* and *CUL4BY*, the breakpoints are less well defined, with sequence identity decreasing over some tens of base pairs.

### Structural rearrangements compared to other species

We generated likely pathways of gene-only rearrangements from the ancestral Y Chromosome to pig, using data from Li et al. (2013a) updated with pig and cattle (Elsik et al. 2009) Y Chromosomes (Supplemental Fig. S12). Global alignments of chromosome content with other available Y Chromosome assemblies shown in Figure 1 are presented as larger versions in Supplemental Figure S8. Although little of the Y sequences align outside genic regions, the comparison highlights which ancestral gene families have become amplified in different lineages, e.g., *HSFY* in pigs and *TSPY* in species including dogs, horses, and humans (Paria et al. 2011; Xue and Tyler-Smith 2011; Li et al. 2013a).

## Discussion

We present here an updated and substantially improved assembly of the pig Chromosome X and a first-generation assembly of sequence from the pig Chromosome Y. These sequences have also allowed us to confirm the strong conservation of the X and recover information on the evolution of the Y Chromosome and how this relates to sex chromosome evolution in other mammals.

### An updated assembly and annotation of the porcine Chromosome X

The picture of mammalian X Chromosomes is one of general structural stability (see Fig. 1). Specific lineages, such as rodents, have many more X rearrangements than others, but these species are characterized by a globally higher number of chromosomal rearrangements (Stanyon et al. 1999). Apparent inversions and translocations in the pig X, relative to the ancestral X, detected in previous builds, are corrected here to an order more reflective of the inferred ancestral X Chromosome. Similar findings may be seen with other mammalian X Chromosomes as the quality of the assemblies improves. It paints a stark contrast to the dynamic and ever-changing mammalian Y Chromosomes that we discuss below.

#### Improved gene annotation of the porcine Chromosome X

The revised gene annotation increases the number of protein-coding genes identified on the pig X to 690, bringing the reported gene content closer to that identified in the X Chromosomes of well-studied species (i.e., humans and mice, with 813 and 940 protein-coding genes, respectively). The majority of X Chromosome genes are shared between species (76% of annotated pig genes shared with human), in accordance with Ohno's law (Ohno 1967b). We have highlighted some specific genes of interest with an updated status from build 10.2 X.

Eleven protein-coding genes present on the human X have been annotated as unitary pseudogenes (also known as loss-of-function genes) on the pig X (Supplemental Table S10). These include: *GUCY2F*, a possible candidate for involvement in X-linked retinitis pigmentosa (Yang et al. 1996); *AWAT1*, an acyl-CoA wax alcohol acyltransferase involved in sebum production (Turkish et al. 2005); *ITIH6*, a trypsin inhibitor; and *RAB41*, a member of the RAS oncogene family (Supplemental Results, section 3).

Other regions of difference lie in the cancer/testis (CT) antigen clusters found in humans and other primates, which are significantly smaller in pig. This is in line with evidence that enlarged CT antigen clusters arose due to a recent amplification in primates (Zhang and Su 2014), perhaps driven by a retrotransposition event. Their potential functions remain unknown, though they may have been involved in primate speciation (Zhang and Su 2014).

Apart from the olfactory receptor gene clusters, we have not found evidence for widespread ampliconic gene families on the pig X. This contrasts with the X Chromosomes of human and mouse, which contain independently amplified gene families, with little overlap between the species (Mueller et al. 2013). Human X Chromosomes contain multiple inverted repeats with high sequence identity, enriched for testis-expressed genes (Warburton et al. 2004). Mice have a greater number of X-ampliconic genes than humans, apparently driven by a genomic conflict between X and Y sequences; the gene *Sly* on mouse Yq represses gene expression from sex chromosomes in spermatids, and the copy number of X genes has increased in response to maintain expression of key genes (Ellis et al. 2011). These examples led to an expectation that this might be a general feature of mammalian X Chromosomes and that the pig X would also contain unique ampliconic testis-expressed genes. However, we have no evidence supporting this—either due to a lack of such genes, the paucity of pig-derived evidence (e.g., ESTs, cDNAs, RNA-seq data), or because ampliconic genes lie within the remaining gaps within the assembly.

### The porcine Chromosome Y

A striking aspect of the Chromosome Y organization is that the known single-copy male-specific genes are found in tight clusters of contigs spanning only 2 or 3 Mb of sequence. This is a pattern observed in some other mammalian Y Chromosomes—for example, mice (Soh et al. 2014), cattle (Elsik et al. 2009), and cats and dogs (Li et al. 2013a). Each lineage appears to have preserved a small region of key ancestral X genes, while the remainder of the chromosome has evolved in a species-specific manner. In contrast, in human and chimpanzee Y Chromosomes (Skaletsky et al. 2003; Hughes et al. 2010), the single-copy material is more widely distributed along the chromosome. Patterns of hybridization from FISH suggest to us that there is additional single-copy sequence on Yq, including near/at the Yq terminus, but we were unable to elucidate the sequences involved.

### Organization of the pig Y

#### Palindromic sequences

A recurring feature of the Y sequences we have assembled is the presence of palindromic regions, each on the order of 120 kb end to end, and reminiscent of ampliconic structures found on the mouse (Soh et al. 2014), human (Skaletsky et al. 2003), and chimpanzee (Hughes et al. 2010) Y Chromosomes. The palindromes on the pig Y have resulted from duplication and inversion of sequences, and at least three such palindromes are present. Two have very high levels of sequence identity; the inverted structure may facilitate the process of gene conversion by allowing the formation of stem–loop structures, as seen in the palindromes on the human Y Chromosome (e.g., Rozen et al. 2003; Hallast et al. 2013). These palindromic sequences are also reminiscent of the “core duplicons” found in humans and great apes (Marques-Bonet and Eichler 2009). In the case of pig, however, there is no evidence for novel gene structures and functional innovation as a result of the duplications.

The first two of these palindromes are in the proximal gene block. One encompasses the two copies of the male-determining gene *SRY*. Multiple *SRY* copies are found in dog (Li et al. 2013a) and some rodent species (e.g., Lundrigan and Tucker 1997; Murata et al. 2010; Prokop et al. 2013), but there has previously been no suggestion of this being the case in the pig. While most other species with multiple *SRY* copies have identifiable sequence differences between the copies in a single individual, there is also a known example in rabbits of a palindrome encompassing *SRY*, with gene conversion maintaining sequence identity (Geraldes et al. 2010). A similar mechanism may maintain the sequence identity between the palindrome arms in pig.

The third palindrome is found in the distal gene cluster. Unlike the previous two palindromes, both breakpoint ends lie within complete transposable elements (TEs). Sequence identity between the palindrome arms is lower around the breakpoints, perhaps indicating that the duplication results from an older event or that homogenizing mechanisms such as gene conversion have been less active. In all three palindromes, the TEs at boundaries are annotated as deriving from within the pig lineage—these are not ancient repeat elements and show the ongoing impact of repetitive content in the genome. Extant suids diverged after ∼10–15 Mya, and the copy number of the genes involved across these lineages remains to be identified.

#### Ampliconic sequences

Most mapped mammalian Y Chromosomes have been found to contain multicopy gene families (e.g., Li et al. 2013a), and the pig is no exception. Outside the palindromes, other sequences have amplified to a much greater extent. There are three gene families of note here, all involved in amplifications in other species and with functions suggesting involvement in spermatogenesis.

The *CUL4B* fragments: Cullin fragments are found proximal to *SRY* and the active *CUL4B* gene and appear to form part of an ampliconic region located toward the centromere. Ubiquitinylation pathways are an important part of gamete development. *CUL4BX* gene defects lead to reduced oocyte survival (Yu et al. 2013) and reduced testis volume in human males (Tarpey et al. 2007).
The *TSPY* fragments: These appear to be interspersed in the region closer to the centromere, but it is not clear how they are arranged. *TSPY* is an ampliconic gene in many mammalian species, from artiodactyls to primates (Xue and Tyler-Smith 2011); the genes are involved in spermatogenesis (Xue and Tyler-Smith 2011) and, in cattle, copy number variation of this gene has been linked with fertility in bulls (Hamilton et al. 2012).
The *HSFY* family: These genes are predominantly found in a block between the proximal and distal low-copy gene clusters and show evidence for recent amplification in the *Sus* lineage to ∼100 copies, with independent amplification in other suid species (Skinner et al. 2015) and further independent amplification in cattle (Chang et al. 2013). *HSFY* is expressed in pig testis and may have a role in spermatogenesis, though biological function remains to be established.

#### Other amplified sequences

Yq is dominated by repeat sequences (as demonstrated by the painting pattern from FISH using several BAC and fosmid clones). These clones are composed almost entirely of sequences related to male-specific (or enriched) repeats described previously for pig (e.g., Mileham et al. 1988), and thus more detailed study is needed to understand the organization of this arm of the chromosome. There is some evidence that related sequences are expressed in testis-derived transcripts; however, expression is not exclusive to this tissue, or to males, with sequence homologies also detected in transcripts from pig uterus. FISH experiments suggest that there is single-copy sequence on Yq, including at or near the Yq terminus, but we were unable to isolate these sequences.

We did not find evidence for exclusively testis-expressed ampliconic sequences. However, ampliconic sequences cause difficulties for assembly that our physical mapping approach was not able to overcome. It is likely that other amplified sequences of biological interest remain to be discovered on the pig Y Chromosome, both on the short and long arms.

#### Comparative chromosome organization and gene order between mammals

Previous work from primates, mouse, cat, and dog has reconstructed a putative ancestral eutherian Y Chromosome (Li et al. 2013a) based on gene order. We incorporated our pig gene order into this and added information from the cattle Y sequence assembly (Supplemental Fig. S12; Elsik et al. 2009). One group of genes stands out from the comparison: *USP9Y-DDX3Y-UTY* is the only ancestral cluster of genes that have retained their proximity to each other in all the studied species. There may be a selective disadvantage to disrupting this organization, as has been proposed for other conserved syntenic blocks in general (Larkin et al. 2009) and for these genes in particular. Both *USP9Y* and *DDX3Y* have been implicated as important in human spermatogenesis, though they may not be essential in all great apes (Tyler-Smith 2008).

#### TRAPPC2P-OFD1Y-GPM6B: a potential transposition from the X

Outside the PAR, there are regions of homologous sequence between the X and Y Chromosomes. Most of the X-Y homologies could be attributed to known X-Y homologous genes or to repetitive sequences, such as endogenous retroviral (ERV) families enriched on the sex chromosomes. An exception was the 90.25-kb region *TRAPPC2-OFD1-GPM6B*, which retains 87% sequence identity across the region and is interrupted only by recent insertion of transposable elements in the Y. The orthologous region has been subject to transposition onto the Y from the X Chromosome in dogs (Li et al. 2013a), and a similar transposition affecting the *RAB9A–SEDL–OFD1Y* genes has occurred in the primate lineage (Chang et al. 2011). Such a large region of homology, including the introns and intergenic regions, argues against mechanisms such as gene conversion, and the proximal end of the region lies within a transposon; consequently, we consider this as evidence suggesting a transposition of this region in the pig (see Supplemental Fig. S1).

*OFD1* is involved in cilia formation, with gene defects affecting multiple tissues (Thauvin-Robinet et al. 2013), and ciliopathies have been implicated in fertility issues (Fry et al. 2014). It is likely that the testis-expressed *OFD1Y* has repeatedly acquired a function in sperm development in different mammalian lineages. Notably, the X copies of *OFD1* and also *CUL4B* have been found to be substantially down-regulated in teratozoospermic men (Platts et al. 2007).

## Conclusion

This work presents an improvement to the pig Chromosome X assembly and gene annotation, and the first assembly of sequence for the pig Chromosome Y. The assemblies we have generated have allowed new insights into the content and evolution of the pig sex chromosomes and provide an important resource for the pig genomics community.

## Methods

### Library construction and sequencing

Chromosome X clones (BAC clones from CHORI 242 library) were sequenced previously under the auspices of the Swine Genome Sequencing Consortium (Groenen et al. 2012).

Phytohaemagglutinin-stimulated peripheral blood culture from a Duroc boar was used to prepare chromosomes for flow sorting. Flow-sorted Y Chromosomes were purified, and 30- to 50-kb-sized fragments were cloned into the pCC1Fos vector (library WTSI_1061: http://www.ncbi.nlm.nih.gov/clone/library/genomic/330/) (Supplemental Methods, sections 1–3). Clones for sequencing were targeted by minimal overlapping clones on a fingerprint contig (FPC) map. The targeted 897 clones for the Y Chromosome were sequenced using a combination of three different sequencing platforms: capillary, Illumina, and 454 (Roche). Clones were assembled using a combination of four assembly scripts to produce de novo assemblies. Manual alignment of clone sequences was used to build the clone map, expanding from clones containing known genes. These contigs were oriented and ordered using fiber-FISH on single DNA-molecule fibers (Supplemental Methods, sections 4, 5).

### Molecular combing and FISH

Single-molecule DNA fibers were prepared by molecular combing (Michalet et al. 1997). Purified fosmid DNA was amplified and labeled as described previously (Gribble et al. 2013). Fluorescence in-situ hybridization followed standard protocols (Supplemental Methods, section 6). Probes were detected with fluorescently conjugated antibodies. Slides were mounted with SlowFade Gold mounting solution containing 4′,6-diamidino-2-phenylindole (Molecular Probes/Invitrogen) and visualized on a Zeiss AxioImager D1 microscope. Digital image capture and processing were carried out using the SmartCapture software (Digital Scientific UK).

### X and Y gene annotation, sequence content, and chromosomal evolution

Manual annotation on the pig X and Y Chromosomes was performed using the Otterlace/Zmap suite of annotation tools (Loveland et al. 2012) following previously established annotation protocols (Harrow et al. 2012; Dawson et al. 2013). The assembled chromosomes were run through an annotation pipeline (Searle et al. 2004), aligning EST, mRNA, and protein libraries against the chromosomes with all annotated gene structures (transcripts) supported by at least one form of this transcriptional evidence. The HUGO Gene Nomenclature Committee (HGNC) (Seal et al. 2011) naming convention was used whenever possible for all pig genes; otherwise, HAVANA naming conventions (http://www.sanger.ac.uk/research/projects/vertebrategenome/havana) were followed.

RepeatMasker (Smit et al. 1996) was used to identify repetitive elements within Y contigs. Targeted resequencing was performed across specific genes to confirm their structure (primers given in Supplemental Table S4). Regions of X-Y homology were identified by comparing the repeat-masked X assembly to all sequenced repeat-masked Y clones (mapped and unmapped) using LASTZ (Harris 2007) with default parameters. Evolutionary analyses between X and Y gene pairs were conducted using the Nei-Gojobori model (Nei and Gojobori 1986) in MEGA5 (Tamura et al. 2011). For each pair, positions containing gaps and missing data were eliminated. Reconstruction of ancestral Y Chromosome organizations was performed using the Multiple Genomes Rearrangement (MGR) program (Bourque and Pevzner 2002) to calculate optimal rearrangement pathways between each species, as previously described (Skinner and Griffin 2012). Full information is given in Supplemental Methods, section 7.

### Gene expression

RT-PCR was used to confirm expression status of selected genes in five tissues (brain, liver, kidney, side muscle, testis), obtained from the same boar from which blood cultures were derived. Samples were taken from tissues stored in RNAlater (Qiagen) and homogenized in TRIzol. Nucleic acids were extracted with phenol-chloroform and DNase I treated. RNA was precipitated with isopropanol and stored at 1µg/µl in ddH_2_O at −80°C. RT–PCR was carried out using a OneStep RT–PCR kit (Qiagen) on 25 ng of total RNA (Supplemental Methods, section 8). Primer sequences are given in Supplemental Table S13.

### Copy-number estimation of *SRY* by qPCR

Primers were designed to amplify a 1447-bp region across the majority of the *SRY* ORF and UTRs (F:TAATGGCCGAAAGGAAAGG; R:TGGCTAATCACGGGAACAAC), and products were generated using a MyTaq Red kit (Bioline) using the following profile: 95°C for 3 min, 35 cycles of 95°C/53°C/72°C for 15 sec/15 sec/2 min, with a final extension of 72°C for 10 min. Two female Duroc gDNAs were spiked with dilutions of the purified *SRY* product to give a standard curve of four copies *SRY* per genome to 0.25 copies per genome (assuming diploid genome size of 6 Gb) (Animal Genome Size Database; Gregory 2006). qPCR was performed using a SYBR-FAST qPCR kit (Kapa Biosystems) on the spiked females and on five male Duroc gDNAs with primers for *SRY* and the autosomal (SSC10) gene *NEK7* (Supplemental Table S6). Annealing temperature was optimized at 57°C. Cycling conditions were 95°C for 3 min, followed by 40 cycles of 95°C/57°C/72°C for 10 sec/20 sec/30 sec. The fluorescent signal threshold crossing point (*C*_t_) was normalized to the average signal from *NEK7* to produce a normalized Δ*C*_t_. The data from spiked female gDNA was used to construct a standard curve relating *SRY* signal to *NEK7* signal; from this, an estimate of the absolute *SRY* copy number in the male gDNA samples was produced (see Supplemental Methods, section 9).

## Data access

All sequence and annotation is available via the Vega Genome Browser (http://vega.sanger.ac.uk/index.html), and the complete chromosomal assemblies can be accessed by FTP (ftp://ftp.sanger.ac.uk/pub/vega/pig/). The pseudoautosomal region of X/Y homology between the X and Y Chromosomes is represented on the X Chromosome only in Vega and Ensembl. It is marked as an assembly exception in both chromosomes, but the underlying genomic sequence and annotation is that of Chromosome X. Only the unique regions of Chromosome Y are stored and annotated. The complete Y Chromosome is represented by filling the “gaps” with the PAR regions from the X Chromosome. Raw sequence data have been submitted to the European Nucleotide Archive (ENA; http://www.ebi.ac.uk/ena/) under accession number ERP001277.

## Supplementary Material

Supplemental Material
