# Supplementary material for: The pig X and Y Chromosomes: structure, sequence, and evolution
Source: Genome Res. 2016 Jan;26(1):130–9. doi: 10.1101/gr.188839.114 (PMC4691746; doi:10.1101/gr.188839.114)
Supplement: Supplemental Material [file supp_26_1_130__index.html]

The pig X and Y Chromosomes: structure, sequence, and evolution — Supplemental Material 

# The pig X and Y Chromosomes: structure, sequence, and evolution

## Supplemental Material

**Files in this Data Supplement:**

- Supp Material.docx
- Supp Tables.xls
