# Supplementary material for: The pig X and Y Chromosomes: structure, sequence, and evolution
Source: Genome Res. 2016 Jan;26(1):130–9. doi: 10.1101/gr.188839.114 (PMC4691746; doi:10.1101/gr.188839.114)
Supplement: Supplemental Material [file supp_gr.188839.114_SuppMaterial.docx]

**Supplementary Material**

**Skinner et al: The pig X and Y chromosomes: structure, sequence and evolution**

**Large supplementary tables are provided in a separate Excel file:**

Table S1 – Ordered Y clone list with gene content

Table S2 - Ordered X clone list

Table S3 – Regions of XY homology

Table S4 – Expression status of Y genes

Table S5 – Gene annotation of the pig X

Table S6 – Gene annotation of the pig Y

Table S7 – Genes on the pig X not found on human X

Table S8 - Unitary pseudogenes on the pig X

| **Supplementary Methods** | 3 |
| --- | --- |
| *1) Pig blood culture* | 3 |
| *2) Flow Cytometric Analysis and Sorting* | 3 |
| *3) Library construction* | 4 |
| *4) Clone sequencing* | 4 |
| *5) Assembly of contigs* | 7 |
| *6) Preparation of single DNA-molecule fibres by molecular combing and fibre-FISH* | 8 |
| *7) Annotation and chromosomal evolution* | 9 |
| *8) Gene expression* | 13 |
| *9) Copy number estimation of SRY by qPCR* | 14 |
| *10) Optical mapping* | 15 |
|  |  |
| *Supplementary Methods Tables* |  |
| Table S9 - Clone sequencing statistics | 7 |
| Table S10 - N50 values calculated with different N numbers | 8 |
| Table S11 - Primers for validation of sequences for OFD1Y and CUL4BY on cDNA | 11 |
| Table S12 - Accessions for Y chromosome assemblies for cross-species alignments | 12 |
| Table S13 - Primers for RT-PCRs | 13 |
| Table S14 - Primers for qPCRs | 15 |
|  |  |
| **Supplementary Results and Figures** | 23 |
| 1) Synonymous substitution rates in XY-homologous genes | 23 |
| Table S15 - synonymous substitution rates across Y gene coding regions | 24 |
| 2) OFD1 region transposition | 25 |
| Figure S1 – OFD region: X versus Y | 26 |
| Figure S2 - Gene expression | 29 |
| Table S16 - Sequence divergence across OFD1 region | 31 |
| 3) Gene content differences on the X chromosome between pig and human | 32 |
| 4) Other supplementary figures |  |
| *a) Physical ordering of clones and sequences* |  |
| Figure S3 - 10.2X vs our X dotplot | 34 |
| Figure S4 - Example of sequence improvement: olfactory cluster | 35 |
| Figure S5 – Detailed Y contigs | 36 |
| Figure S6 – Unplaced Y contigs structure | 37 |
| Figure S7 – FISH of unplaced Y contigs on Yp towards the centromere | 38 |
| *b) Cross-species comparisons of X and Y* |  |
| Figure S8 - Dotplots of pig X and Y against other species (as Figure 1) |  |
| a - Dog (*Canis familiaris*) | 39 |
| b - Cat (*Felis catus*) | 39 |
| c - Cow (*Bos taurus*) | 40 |
| d - Sheep (*Ovis aries*) (X only) | 40 |
| e - Mouse (*Mus musculus*) | 41 |
| f - Rat (*Rattus norvegicus*) (X only) | 41 |
| g - Rabbit (*Oryctolagus cuniculus*) (X only) | 42 |
| h - Human (*Homo sapiens*) | 42 |
| i - Chimpanzee (*Pan troglodytes*) | 43 |
| *c) The pig Y SRY region* |  |
| Figure S9 – SRY region: order of inversions | 44 |
| Figure S10 – Copy number of SRY | 45 |
| *d) Other Y chromosomal rearrangements* |  |
| Figure S11 – USP9Y region | 46 |
| Figure S12 – gene order rearrangements on mammalian Y chromosomes | 47 |
| Figure S13 - Other FISH results | 48 |
|  |  |
| **Supplementary References** | 49 |
|  |  |
| **Acknowledgements** | 51 |

# **Supplementary Methods**

*1) Pig blood culture*

Whole blood from a Duroc boar was collected into sodium heparin-coated tubes (BD). The anti-coagulated blood was layered into the upper chamber of the prefilled ACCCUSPINTM System-HISTOPAQUE©-1077 (Sigma) and centrifuged at 1000g for 10 min. The nucleated cells (mostly lymphocytes) at the interface were removed and washed once with HBSS, and then cultured in PB-MAXTM Karyotyping Medium (Invitrogen) for 72h before harvest for metaphase chromosomes.

*2) Flow Cytometric Analysis and Sorting*

Peripheral blood mononuclear cell (PBMC) from the blood of a boar was prepared as described above. After 72h of culture in phytohemagglutinin, the stimulated culture was blocked at metaphase with 0.1µg/ml colcemid for 5hr prior to chromosome preparation.

Chromosomes were prepared using a modified polyamine isolation buffer as described previously (Ng and Carter 2006). The chromosome preparation was stained overnight with Hoechst 33258 (HO) and Chromomycin and analysed and purified using a flow sorter (Mo-Flo^®^, Beckman Coulter) configured for high speed sorting. Data for HO fluorescence, Chromomycin fluorescence, forward scatter and pulse width were collected using HO fluorescence as the trigger signal. A region was created on a plot of linear FSC versus linear pulse width to exclude clumps and debris, and bivariate plots of Hoechst versus Chromomycin fluorescence were gated on this region. The stained chromosome suspension was flow sorted at a data rate of 4000-5000 events/s, with optimal setting of the sheath pressure to 60 psi and the drop frequency to 95kHz using a 70µm cytonozzle tip on a high purity sort mode. The chromosomes were flow sorted into sterile 1ml eppendorf tubes and the DNA was purified and extracted as described previously (Gribble et al. 2004).

*3) Library construction*

Flow sorted chromosomes (approx. 300ng) were centrifuged at 13000rpm for 15 min at RT, washed in 70% ethanol followed by 100% ethanol, and then air dried for 1 hour. DNA was dialysed twice in ½ x TE for 1 hour to remove salts and then ethanol precipitated and re-suspended in 34ul T0.1E. DNA was end-repaired using the Epicentre End-It kit following the manufacturer’s protocol, then purified by phenol chloroform extraction. DNA was resolved on CHEF gel using 5 x TBE with the instrument set to 4.5v/cm, 1s-7s switch time for 17h. Resultant current was 110-120mA. A 30-50kb fragment was excised and DNA extracted from the gel slice using Epicentre Gelase kit followed by phenol chloroform extraction, ethanol precipitation with the resultant pellet suspended in 5µl T0.1E. The DNA was ligated into pCC1Fos (Epicentre) using Roche T4 ligase at 12-14°C for 16 hours, then heat inactivated at 70°C for 10mins. 1µl ligation was packaged with 100µl MaxPlax Lambda packaging extracts (Epicentre) as manufacturer’s instructions and stored at 4°C. 6 x 100µl of the packaged phage was mixed with 10ml EPI300 (Epicentre) cells (OD 1.02), incubated at 37°C for 25mins, topped up to 50ml with LB and incubated for a further hour at 37°C. Cells were centrifuged at 3000rpm for 10mins, resuspended in 1ml LB, and then added to 330µl 80% glycerol. Cells were spread on plates in 50µl aliquots.

*4) Clone sequencing*

The 964 chromosome X clones were sequenced previously under the auspices of the Swine Genome Sequencing Consortium project (Groenen et al. 2012). These were a set of overlapping BAC clones selected from a fully fingerprinted CHORI 242 library (Humphray et al. 2007).

The targeted 897 clones for the Y chromosome were sequenced using a combination of 3 different sequencing platforms: Capillary; Illumina and 454. Clone DNA was sheared and subcloned into pUC19 vector with a 2-4kb insert size and sequenced with BigDye terminator chemistry on the ABI3700 and ABI3730 automated sequencers (Quail et al. 2011).

Multiplexed standard Illumina libraries were prepared from single pig clone DNA preps following standard protocols (Quail et al. 2009) with 450-550bp inserts and sequenced on the Illumina Genome Analyzer II and HiSeq 2000 machines following the manufacturer’s standard cluster generation and sequencing protocols. Paired end sequence runs were performed to provide draft sequences for the Pig X and Y chromosomes, using the 76bp paired read length on the GAII platform and a 100bp paired on the HiSeq platform as sequencing capabilities evolved in the course of the project. Raw sequence data is submitted to the public data repository, ENA<http://www.ebi.ac.uk/ena/>, under accession number ERP001277.

In addition, a few clones were sequenced, using Multiplexed Identifiers (MIDs), on the Roche 454 GS-FLX+ Titanium Genome Sequencer with a 3kb insert as per manufacturer’s protocol.

The Capillary clones were assembled using phrap2gap (Dear et al. 1998). The Genome Assembly Program GAP4 (Bonfield et al. 1995) was used to view and edit the capillary sequence assemblies. The Illumina and 454 sequenced clones were assembled using a combination of four assembly scripts to produce *de novo* assemblies: Illumina Clone Assembly System (iCAS; http://sourceforge.net/projects/icas/); Assembly by Short Sequences (ABySS) which is a *de novo*, parallel paired end sequence assembler (Simpson et al. 2009), the Velvet optimizer, a *de novo* assembler for short read sequencing (Zerbino 2010), and the 454/Roche Newbler assembly program. The data were viewed and edited using the Genome Assembly Program GAP5 (Bonfield and Whitwham 2010).

Chromosome X and Y clones were selected for further sequence improvement so as to reach a *Finished* standard independent of the sequencing platform used to generate the raw data (Chain et al. 2009). This was facilitated by closing down all gaps, poor quality regions and correcting misassemblies using Chain termination (Sanger) sequencing methods. All the remaining sequence gaps and problem regions within each clone were bridged by end-sequencing Polymerase Chain Reaction (PCR) products sequenced with BigDye terminator chemistry on the ABI3700 capillary sequencers or using read pair information in capillary specific clones). Specifically for the Illumina clones further computational improvement programs were used: Iterative Mapping and Assembly for Gap Elimination (IMAGE) was used to shorten or close down as many sequence gaps as possible through an iterative process utilizing Illumina paired-end (Tsai et al. 2010). This was followed by the use of the Iterative Correction of Reference Nucleotides program (iCORN) to detect and correct base errors, using an iterative algorithm utilising paired-end Illumina data (Otto et al. 2010). The final clone assemblies irrespective of their sequencing platforms were confirmed by restriction digest information and independently checked to ensure the minimum criteria had been met.

Clones that had additional work performed beyond the initial shotgun sequencing draft assembly but were not able to achieve the *Finished* status were improved to the community defined genome standard of *Improved High Quality Draft* (Chain et al. 2009) where no discernable misassemblies exist and that some form of computational gap resolution has taken place to reduce the number of contigs. All contigs have been ordered and orientated where possible and the clone assembly was guided by restriction digest information. Undetectable misassemblies may still exist, particularly in repetitive regions. Low-quality regions and potential base errors may also be present. All repeats were checked and assembled correctly where possible or broken if found to be incorrect.

*Finished* clones were released as phase 3 complete sequences: clones are in one contiguous piece of DNA and of *Finished* quality (Chain et al. 2009). Otherwise, clones were released as phase 2 sequences: sequences are unfinished but the order and orientation of the pieces are more often than not known, however, the length of the gaps between pieces may or may not be know. An unfinished sequence in one piece can also be released as Phase 2. Phase 2 relates to the community defined Improved High Quality Draft standard (Chain et al. 2009). [Phase 1 indicates an unfinished sequence containing gaps in which the order and relative orientation of the pieces are not known. This phase relates to the community defined genome standard of High Quality Draft (Chain et al. 2009). There are no Phase 1 clones]. All clone sequences are submitted to EMBL [http://www.ebi.ac.uk/embl/.](http://www.ebi.ac.uk/embl/)

| **Clone Details** | **Chromosome X** | **Chromosome Y** |
| --- | --- | --- |
| Number of clones | 964 | 897 |
| Clones completed to phase 3 | 901 | 167 |
| Clones completed to phase 2 | 63 | 730 |
| Total number of contigs | 5 | 680 |
| Number of gaps | 13 | 709 |
| Total length of *Finished* clones without overlap (bp) | 123,752,255 | 6,039,935 |
| % of chromosome *Finished* based on chromosome size estimates of 128Mb for chr X and 50Mb for chr Y | ~ 96.7% | ~12% |
| Total length of *Finished* and unfinished clones without overlap | 128,635,921 | 29,953,871 |

**Table S9**: Clone sequencing statistics

*5) Assembly of contigs*

Manual alignment of clone sequences was used to build the clone map, expanding from clones containing known genes. These small contigs were oriented and ordered using fibre-FISH on single DNA-molecule fibres. Final sequence contigs were assembled based on this map using GAP5 (Bonfield and Whitwham 2010). N50 values were calculated splitting contigs on runs of 100 or more “N”s. These values were robust to down to a single “N”, and up to 101 or more "N"s (i.e. keeping unfinished clones in one piece).

| **Number of “N”s to call split** | **X: N50** | **Y: N50** |
| --- | --- | --- |
| 1+ | 4824757 | 66937 |
| 100+ | 4824757 | 66965 |
| 101+ | 5434259 | 91362 |

**Table S10:** N50 values calculated with different N numbers

*6) Preparation of single DNA-molecule fibres by molecular combing and fibre-FISH*

Single-molecule DNA fibres were prepared by molecular combing (Michalet et al. 1997) according the manufacturer’s instructions (Genomic Vision) using fibroblast cells of a Duroc boar. Briefly, the cells were embedded in a low-melt-point agarose plug (1 million cells per 90µl plug), followed by proteinase K digestion, washing in 1×TE (10mM Tris, 1mM EDTA, pH8.0) & beta-agarase digestion steps. The DNA fibres were mechanically stretched onto saline-coated coverslips using a Molecular Combing System (Genomic Vision). To make FISH probes, purified fosmid DNAs were first amplified using a GenomePlex® Whole genome Amplification (WGA) kit (Sigma-Aldrich) following the manufacturer’s protocols, then labelled using a WGA reamplification kit (Sigma-Aldrich) using a custom-made dNTP mix as described before (Gribble et al. 2013). For the fibre-FISH approximately 500 ng of labelled DNA from each probe and 4 μg of porcine Hybloc DNA (Applied Genetics Laboratories) were precipitated using ethanol, then resuspended in a mix (1:1) of hybridisation buffer [containing 2×SSC, 10% sarkosyl, 2M NaCl, 10% SDS and blocking aid (Invitrogen)] and deionised formamide (final concentration 50%). Coverslips coated with combed DNA fibres were dehydrated through an 70%, 90% and 100% ethanol series and aged in 100% ethanol at 65°C for 30 seconds, followed by denaturation in an alkaline denature solution (0.5M NaOH, 1.5M NaCl) for 1-3 minutes, three washes with 1xPBS (Invitrogen) and dehydration through an 70%, 90% and 100% ethanol series. The probe mix was denatured at 65°C for 10 minutes before being applied onto the coverslips and the hybridisation was carried out in a 37°C incubator overnight. The post-hybridisation washes consisted of two rounds of washes in 50% formamide/2×SSC (v/v), followed by two additional washes in 2×SSC. All post-hybridisation washes were done at 25°C, for 5 minutes each. Digoxigenin-11-dUTP (Roche) labelled probes were detected using a 1:100 dilution of monoclonal mouse anti-dig antibody (Sigma-Aldrich) and a 1:100 dilution of Texas Red-X-conjugated goat anti-mouse IgG (Molecular Probes/Invitrogen); DNP-11-dUTP (PerkinElmer) labelled probes were detected using with a 1:100 dilution of Alexa 488-conjugated rabbit anti-DNP IgG and 1:100 dilution of Alexa 488-conjugated donkey anti-rabbit IgG (Molecular Probes/Invitrogen); biotin-16-dUTP (Roche) labelled probes were detected with one layer of 1:100 dilutions of Cy3-avidin (Sigma-Aldrich). After detection, slides were mounted with SlowFade Gold® mounting solution containing 4’,6-diamidino-2-phenylindole (Molecular Probes/Invitrogen). Images were visualised on a Zeiss AxioImager D1 microscope. Digital image capture and processing were carried out using the SmartCapture software (Digital Scientific UK).

*7) Annotation and chromosomal evolution*

Manual annotation on the pig X and Y chromosomes was performed using the Otterlace/Zmap suite of annotation tools (Loveland et al. 2012) following well established annotation protocols as used to create the reference GENCODE gene sets for human and mouse (Harrow et al. 2012) as well as for the porcine immunome (Dawson et al. 2013).

The assembled chromosomes were run through an annotation pipeline (Searle et al. 2004), aligning EST, mRNA and protein libraries against the chromosomes with all annotated gene structures (transcripts) supported by at least one form of this transcriptional evidence. The HUGO Gene Nomenclature Committee (HGNC) (Seal et al. 2011) naming convention was used whenever possible for all pig genes, else HAVANA naming conventions (<http://www.sanger.ac.uk/research/projects/vertebrategenome/havana>) were followed.

ENSEMBL RNAseq models for the Sscrofa 10.2 X chromosome assembly, built via the ENSEMBL RNAseq pipeline (http://www.ensembl.org/info/genome/genebuild/2012_04_sus_scrofa_genebuild.pdf) with RNAseq data provided by the Swine Genome Sequencing Consortium (SGSC), were remapped onto our new X chromosome assembly. The RNAseq data used by ENSEMBL comprised a mixture of single and paired end data from samples including: a pool of 10 tissues, alveolar macrophages, male gonad, whole blood, placenta and testis. Any novel RNAseq models were assessed under HAVANA’s annotation protocols and annotated and added to the manually annotated gene set as appropriate.

All annotation is available via the Vega genome browser (<http://vega.sanger.ac.uk/index.html>). The pseudoautosomal region of X/Y homology between the X and Y chromosomes is represented on the X chromosome only in Vega and Ensembl. It is marked as an assembly exception in both chromosomes, but the underlying genomic sequence and annotation is that of the X chromosome. Only the unique regions of the Y chromosome are stored and annotated. The complete Y chromosome is represented by filling the 'gaps' with the PAR regions from the X chromosome.

RepeatMasker (Smit et al. 1996) was used to identify repetitive elements within our contigs. Gene content and structure was identified both by gene prediction program GeneMark (<http://exon.gatech.edu/GeneMark/>) and manual comparison to known Y genes using BLAST. Targeted resequencing of cDNA from testis was performed for CUL4B and ODF1 to confirm their structure (primers given in supplementary table S4). All PCR products were subcloned, and sequenced to distinguish Y derived products from X homologues. Overlapping fragments allowed reconstruction of the transcripts, which were validated by realignment against Y genomic clones to confirm the presence of consensus splice junctions at the exon boundaries.

| Gene fragment | Forward | Reverse | Product  size (bp) |
| --- | --- | --- | --- |
| OFD1Y_1 | AACCGCAGAGAAAGCAAGTG | TCTAACTGTGGGTGCTGAGG | 778 |
| OFD1Y_2 | TTTCCGGAAAGTGGTTTGGC | AGTCCTCCTTCAGTTCCAGC | 802 |
| OFD1Y_3 | TTGCTGAGAGGGAGAGAAGC | CCATGGTGTAACTGTGCCTG | 939 |
| OFD1Y_4 | AAACGCTTTGAGACAGGACG | AAGTGGTTTCGGATCAACGC | 814 |
| OFD1Y_4A | CAGGCACAGTTACACCATGG | ATTGGGCTGGAAAGTGAGTG | 532 |
| OFD1Y_5 | TCTCTCCCCAAAGCGAGAAG | CACCAAGCAGtTACCAAGAG | 796 |
| CUL4BY_1 | CGCATCACCTTCATCTACTGC | AGCTTCCTTCCCTCTGATGC | 789 |
| CUL4BY_2 | CTGAAGCAGCTTTGTGAAAACC | TCCAGAAGTTCTTGCACCATTG | 785 |
| CUL4BY_3 | TGCATCAGAGGGAAGGAAGC | CCACTGAAGTTTCCGACCAC | 979 |
| CUL4BY_4 | TCCAGTTGCCTCCAGAGATG | AACAGGACCACGAGTAACCC | 723 |

**Table S11 - Primers for validation of sequences for OFD1Y and CUL4BY on cDNA**

Regions of XY homology were identified by comparing the VEGA repeatmasked X assembly (<http://vega.sanger.ac.uk/Sus_scrofa/Location/Chromosome?r=X-WTSI>) to all sequenced repeatmasked Y clones (mapped and unmapped) using LASTZ (Harris 2007). Unannotated repetitive content enriched on the sex chromosomes was minimised by performing an X-X self alignment, and subtracting multiply-hit regions from the X-Y comparison. Candidate regions of 1kb or longer were further analysed using BLAST for similar sequences in the NCBI NT and EST databases, and the VEGA X gene annotations.

Evolutionary analyses between X and Y gene pairs were conducted using the Nei-Gojobori model (Nei and Gojobori 1986) in MEGA5 (Tamura et al. 2011). Reconstruction of ancestral chromosome organisations was performed using the Multiple Genomes Rearrangement (MGR) program (Bourque and Pevzner 2002) to calculate optimal rearrangement pathways between each species, as previously described (Skinner and Griffin 2012).

The previous X assembly was compared with the current X assembly, and our X and Y sequences were compared with other representative mammalian X and Y chromosomes (list). X and Y chromosome sequences for other mammalian genomes were downloaded from the Ensembl 77 FTP site (ftp://[ftp.ensembl.org/pub/release-77/fasta/](http://ftp.ensembl.org/pub/release-77/fasta/)). Where Y sequence was unavailable from Ensembl, accessions are given in Table S13.

| Species | Y accession |
| --- | --- |
| Cattle | Genbank:CM001061.2 |
| Cat | Genbank:KP081776 |
| Dog | Genbank:KP081775 |
| Rhesus macaque |  |

**Table S12 - Accessions for assemblies used in comparative alignments**

The sequences were aligned to the pig sequences using lastz (v 1.02.00) with either default parameters (yellow lines) or very stringent options (--notransition --step=30 --seed=match12 --exact=50 --matchcount=1000 --masking=3; blue lines). The output in axt format was used to bull chains with the axtChain software (git://genome-source.cse.ucsc.edu/kent.git; 26 Nov 2014) using the loose linear gap model (-linearGap=loose) to build longer structures. Lastly, chains shorter than 1kbp are discarded. If a chain contains a gap longer than 1kbp, it is split at the gap.

For comparing the new X assembly to the version in Sscrofa10.2, we use the aforementioned stringent parameters only. Blue lines correspond to chains that are at least 10Kbp in length while grey lines show those that are at least 1Kbps long. As before, chains containing long gaps (10Kbp and 1Kbp respectively) are split. The code and command line details are available at:<https://github.com/jherrero/oxford-plots>.

Regions reported as inverted between the Tibetan wild boar and the 10.2 X were taken from Li et al. (2013b).

*8) Gene expression*

RT-PCR was used to confirm expression status of selected genes in five tissues (brain, liver, kidney, side muscle, testis), obtained from the same boar from which blood cultures were derived. Samples were taken from tissues stored in RNAlater (Qiagen) and homogenised in Trizol. Nucleic acids were extracted with phenol-chloroform and DNase treated. RNA was precipitated with isopropanol and stored at 1µg/µl in ddH_2_O at -80°C. RT–PCR was carried out using a OneStep RT–PCR kit (Qiagen) on 25 ng of total RNA. Primer sequences are given in supplementary table S5. Where possible, at least one primer is Y specific. When X/Y primers were used all products were subcloned and sequenced to distinguish X from Y sequence. RT-PCR used primers with more mismatches to improve Y selection over X

| Gene fragment | Forward | Reverse | Product  size (bp) |
| --- | --- | --- | --- |
| CUL4BY_cA | ACAGATGCCTTGACTAAACCCCC | AATTCAAGCCCCATGTCCCAA | 482 |
| CUL4BY_cB | TATGCATCAGAGGGAAGGAAGC | ACTTGAACTCCACATTCTACCC | 278 |
| CUL4BY_cC | TGTAGAAAAGCAGCTTCTTGGTG | TTGTTACCTGCACGAAGTTTTGAA | 399 |
| CUL4BY_cD | TTTCTACTTAAGCAAGCACAGTGG | GAGGACTCTAGCTCTCCCACA | 250 |
| OFD1Y_B | TTCCGGAAAGTGGTTTGGCT | TCCGGAAATCAGTCAATTCCTTCT | 459 |
| OFD1Y_C | TGAAGCCCTCATTTCTCGGG | TTCAGCTCCTGCCTCCAAAG | 239 |
| TMSB4Yc1 | CCACTGCCTTCAAAAGAAACGA | TACAGCCCTCAGGACATCAC | 354 |
| TXLNGYc1 | TACAGCTTGTGGTTGGCACA | GTGGCCCTCTGAATCTTGCT | 287 |
| ZFY | CTGATCCTCTGACAGCCGAC | TCTCAGGCTCACTCTCCACA | 330 |
| ZRSR2Y | AATAGTGGGTGGGGAAATGACG | CTGTGGGTGGTTCTGGGTTTT | 385 |
| TRAPPC2Y* | GTGGAATCTCGCGAGGGTAG | ACCCCATTGTAGTTTCACTGT | 810 |

**Table S13 - Primers for RT-PCRs** *Designed to link 5’ sequences found in clone WTSI_1061-7J22 to the 3’ sequence homology to the X gene seen in WTSI_1061-77N24. However, there is no evidence that the equivalent 3’ end is expressed from Y, although 5’ related sequences are seen in 4 ESTs.

*9) Copy number estimation of SRY by qPCR*

Primers were designed to amplify a 1447bp region across the majority of the *SRY* ORF and UTRs (F:TAATGGCCGAAAGGAAAGG; R:TGGCTAATCACGGGAACAAC), and products were generated using a MyTaq Red kit (Bioline) using the profile 95°C for 3mins, 35 cycles of 95°C/53°C/72°C for 15s/15s/2min with a final extension of 72°C for 10min. Two female Duroc gDNAs were spiked with dilutions of the purified *SRY* product to give a standard curve of 4 copies *SRY* per genome to 0.25 copies per genome (assuming diploid genome size of 6Gb; Animal Genome Size Database; Gregory 2006). qPCR was performed using a SYPR-FAST qPCR kit (Kapa Biosystems) on the spiked females and on 5 male Duroc gDNAs with primers for *SRY* and the autosomal (SSC10) gene *NEK7* (supplementary table S6). Annealing temperature was optimised at 57°C. Cycling conditions were 95°C for 3mins, followed by 40 cycles of 95°C/ 57°C/72°C for 10s/20s/30s. The fluorescent signal threshold crossing point (Ct) was normalized to the average signal from *NEK7* to produce a normalised ΔC_t_. The data from spiked female gDNA was used to construct a standard curve relating *SRY* signal to *NEK7* signal; from this, an estimate of the absolute *SRY* copy number in the male gDNA samples was produced.

| Gene | Forward | Reverse |
| --- | --- | --- |
| *SRY* | TACAGAAGCCGAAAAGCGCC | CTTGCGACGAGGTCGGTATTT |
| *Nek7 (SSC10)* | GGGATGAATCAGAGCAATCAC | TGAAAGGGCAGAGAAAATCC |

**Table S14 - Primers for qPCRs**

*10) Optical mapping*

Whole genome mapping (optical mapping) enables the construction of whole-genome restriction enzyme maps through the assembly of single DNA molecules that have been linearised, fixed in position on a positively charged glass surface and subsequently cut with a restriction enzyme. These single DNA molecule restriction enzyme maps are then stained, visualised and the individual component fragments of each molecule accurately sized. The single-molecule maps are subsequently assembled into contigs in a process comparable to shotgun sequence assembly, with the resultant contigs spanning entire chromosomes. High depth of coverage providing a robust map to which sequence contigs can be aligned, ordered, orientated and independently validated.

The primary step in the whole genome mapping (optical mapping) process is the extraction of high molecular weight DNA. For this project cells were embedded in agarose plugs (100ul) to minimise physical shearing of the extracted DNA and treated overnight with Proteinase K 2mg/ml in NDS solution. The plugs were subsequently washed in TE (pH 8), melted, mixed with 100ul of an Agarase solution (4ul Agarase (1000U/ml)/96ul TE) and heated to 42°C for 12 hours, resulting in a high molecular weight genomic DNA sample.

Prior to data collection an appropriate restriction enzyme with which to digest the genome was selected by in silico digestion of the available sequence data using enzyme selection software from OpGen. KpnI was identified as an enzyme which cut the Pig genome suitably, with the primary considerations being that the average fragment size generated would fall between 6kb and 12kb and that there were not a large number of regions of particularly low or excessively high density of restriction sites, which may be problematic in assembly.

Following sample quality controls checks and appropriate dilutions using OpGen QCards, whereby samples are diluted out by eye to an optimal level for data generation (approximately 500pg/ul), 2μL of DNA was applied to MapCards and run on the Argus® system following manufacturer's protocols. Here DNA was flowed down a microfluidic device into channels where the DNA was linearised and fixed in position on the charged mapping surface. The MapCard chambers were then loaded with JOJO™ stain, KpnI restriction enzyme, buffer and antifade. The card was then cycled on the Argus® MCP (MapCard Processing Unit) for approximately 25 minutes, where reagents are flowed over the DNA sample and digestion occurs at 37°C.

The MapCard lanes were then systematically scanned in the Argus Mapping Unit, identifying the restriction pattern of individual molecules (Figure OM1) and accurately measuring the distance between restriction sites (Figure OM2). The resultant dataset consisted of 4,565,916 molecules with an average molecules length of 391.42 Kb and an average restriction fragment size of 14.53Kb.


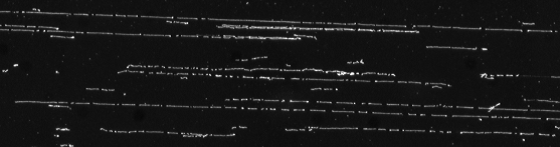


**Figure OM1 -** MapCard lane image post molecule digestion


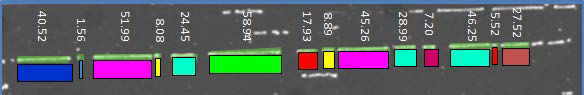


**Figure OM2 -** Molecule fragment sizing

De novo assembly of the X chromosome was completed at OpGen Inc, resulting in an assembly of 5 optical contigs with a total size of 123.76 Mb and an average size of 24.75 Mb.

Whole genome map (WGM) *de novo* assembly takes a bottom-up approach to perform *de novo* super-scaffold assembly. Briefly, it sorts single-molecule restriction maps (SMRM) generated in Argus^TM^ into groups. The sorting process can be realized by comparing SMRMs with available reference DNA sequence scaffolds, taking different SMRM quality category, and/or taking different SMRM size range. Each group of maps then enters a parallel assembly process, which significantly improves the computational efficiency. WGM seeds are assembled from aforementioned process. In the next step, each seed is expanded and re-assembled by utilizing the entire set of SMRMs (beyond the group of maps used for seed assembly) in an iterative manner. Expanded seeds create overlapping regions, which are used to anchor seeds together, and eventually build a super-scaffold. Finally, the super-scaffolds are formed by assembling overlapping seeds.


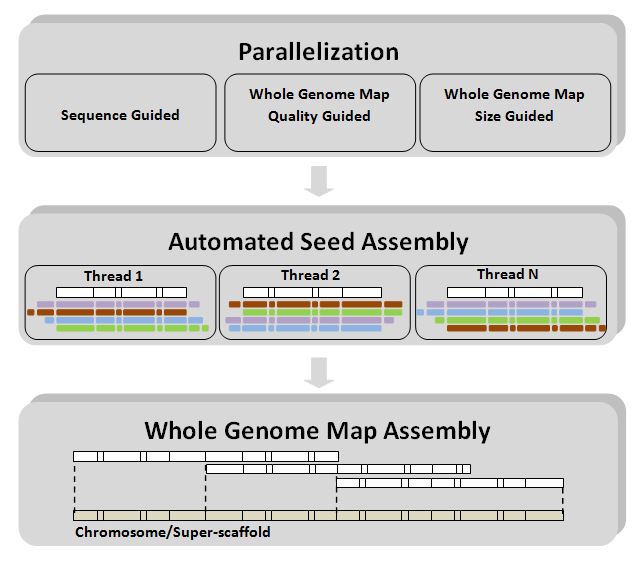


**Figure OM3** - OpGen’s *de novo* assembly approach uses the single molecular restriction maps (SMRM) to *de novo* assemble super-scaffolds. The SMRMs are first sorted into groups. Each group of maps is then assembled into seeds in a parallel fashion. Each seed is further expanded and assembled independently. Finally, the overlapping regions are created by expanded seeds and the super-scaffold is formed by re-assembling overlapped seeds.



Once the assembly process was considered complete a thorough QC review was undertaken to ensure all regions of map assembly were considered robust. This was carried out using the QC module of MapManger™ from OpGen, this software seeks to identify regions of potential missing cut sites, false cuts, mis-assembly and low coverage which once highlighted can be fully reviewed, investigated and where appropriate resolved.

The mapping data generated was subsequently aligned to the sequence assembly in MapSolver™ software and utilised to order and orientate sequence scaffolds, size sequence gaps, accurately identify repeat copy number and independently validate the sequence assembly. Additionally each individual clone sequence was in silico digested and aligned to the map (Figure OM4) enabling the verification of individual clone location, order, orientation and assembly.


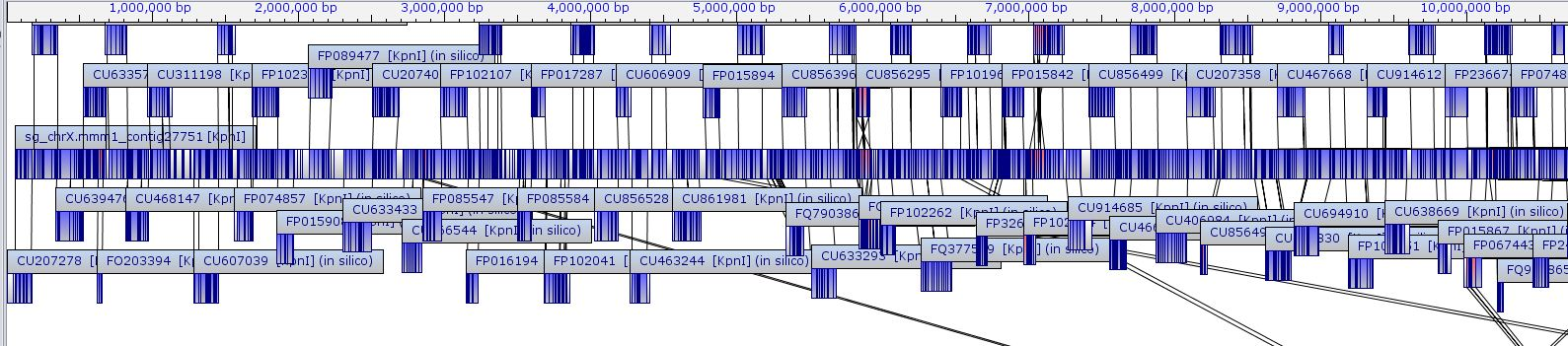


**Figure OM4 -** region of pig chromosome X map and associated clone alignments

This approach enabled the accurate sizing of sequence gaps, in Figure OM5 a gap can be seen in Chromoview between CU914789 and FP312632. By *in silico* digesting these two clone sequences and aligning them to the mapping information (Figure OM6) both the assembly over the region can be confirmed and the sequence gap size accurately identified.


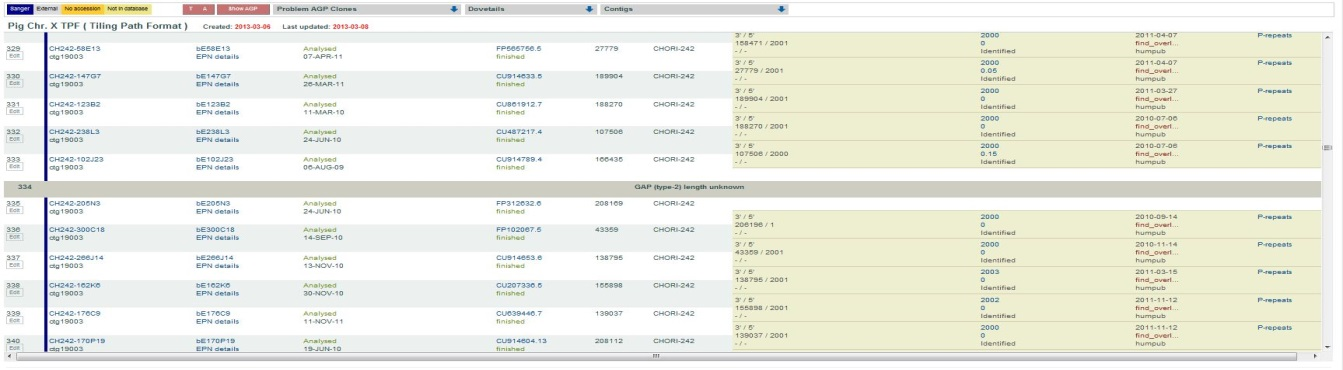


**Figure OM5 -** gap in Chromoview between CU914789 and FP312632


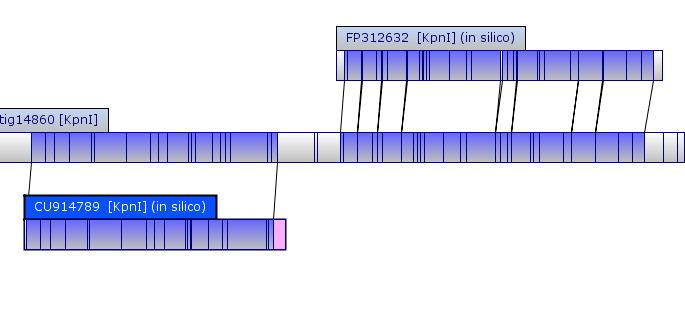


**Figure OM6 -** alignment of clones in Figure OM5 to determine sequence gap size

Together with some larger scale relocation of clones this comparison of map and sequence facilitated the correction of several mis-assemblies contained within single clones. Figure OM7 illustrates alignments before (top) and after (bottom) the correction of FP016027 to the mapping data (centre). Similarly, Figure OM8 again identifies this type of map corrected false inversion, this time contained within clone CU607027.


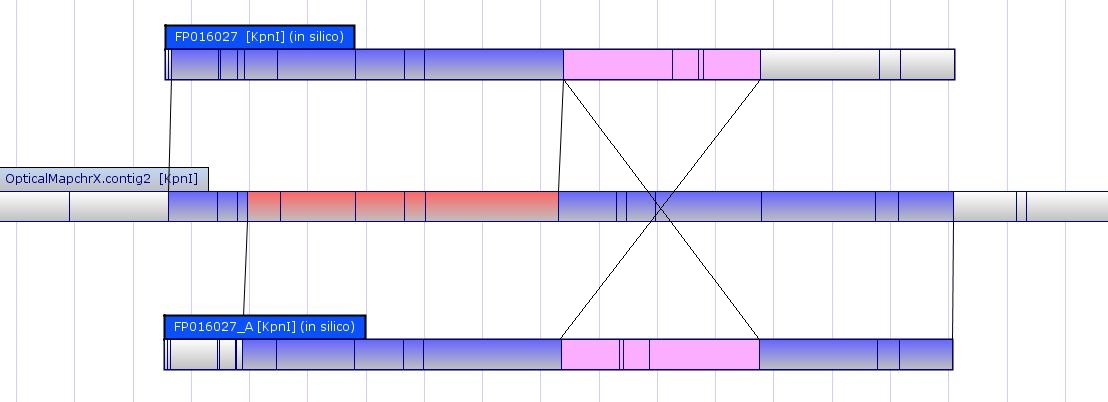


**Figure OM7** - Correcting a clone mis-assembly within FP016027 (CH242-202P13)


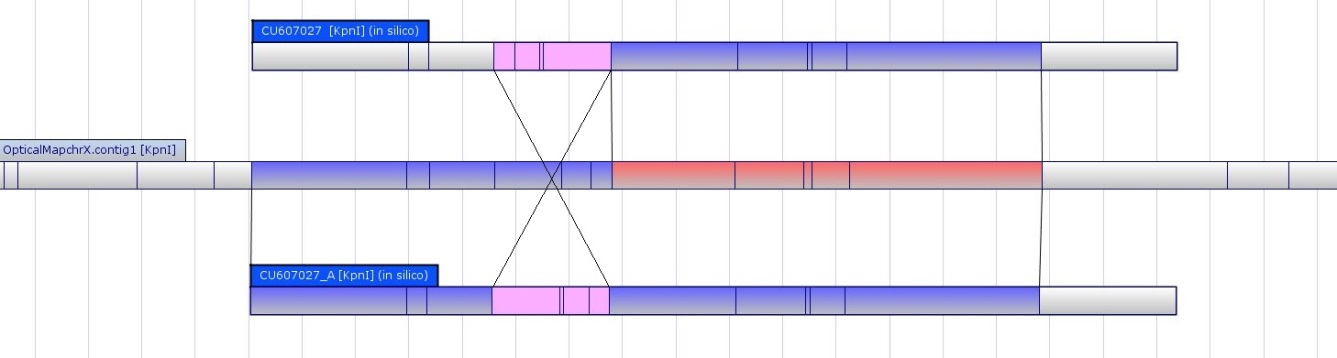


**Figure OM8** - Correcting a clone mis-assembly within CU607027 (CH242-174M23)

In addition to the de novo assembly of Chromosome X the genomic data collected was used to both size gaps and identify potential sequence data relocation with the whole genome. This was achieved by utilising Genome Builder software from OpGen, Genome Builder (Figure 8) works by in silico digesting the existing sequence assembly, identifying the restriction pattern at scaffold ends and then looking for optical data which matches these regions and iteratively extending from the scaffold ends until a hit is found with another scaffold. Thus gaining sequence order, orientation and accurate gap sizing information. The existing Pig sequence assembly was broken at points of >5,000 n’s to allow gap sizing and sequence relocation to occur. This resulted in a sequence input of 3623 scaffolds, utilising this approach we were able to size 2540 of these gaps and identify a number of potential regions where sequence should be relocated to alternative chromosomes.


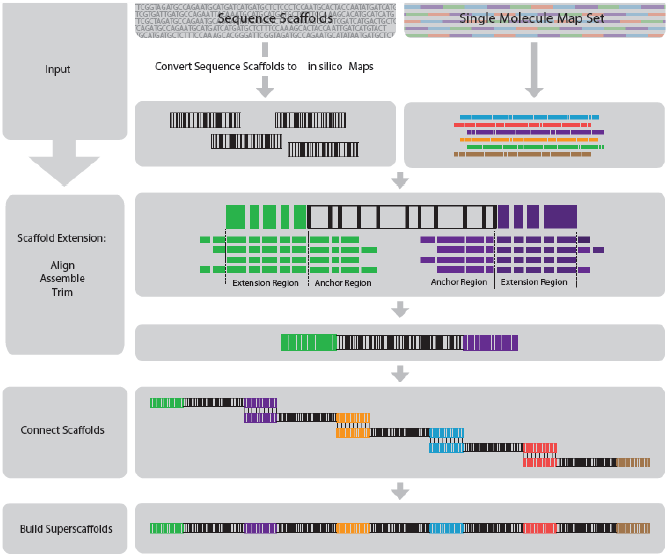


**Figure OM9** - using Genome Builder (OpGen) to construct scaffolds and identify potential sequence data relocation with the whole genome

#

# **Supplementary Results and Figures**

1. **Substitution rates within XY-homologous genes**

We examined rates of synonymous substitution in open reading frames between the X and Y copies of genes conserved on Y chromosomes across mammalian species. Genes from more ancient evolutionary strata as defined in the human X (Bellott et al. 2014) have higher number of synonymous substitutions per synonymous site. The low K_S_ of the *ZFX/Y* and *AMELX/Y* genes is consistent with previous description of ongoing gene conversion in other mammalian species (e.g. Slattery et al. 2000; Pamilo and Bianchi 1993).

| **Gene pair** | **X accession** | **Y accession** | **Aligned length** | **K_S_** | **K_A_** | **K_A_/K_S_** | **Stratum**  **(Bellott et al. 2014)** |
| --- | --- | --- | --- | --- | --- | --- | --- |
| *RBMX/Y* | OTTSUST00000003261 | OTTSUST00000013724 | 1185 | 0.826 | 0.112 | 0.136 | 1 |
| *SOX3-SRY* | *OTTSUST00000012290* | OTTSUST00000006277 | 447 | 2.094 | 0.147 | 0.070 | 1 |
| *HSFX/Y* | *OTTSUST00000013048* | OTTSUST00000013621 | na | na | na | na | 1 |
| *UBA1X/Y* | OTTSUST00000005989 | OTTSUST00000012986 | 3177 | 0.686 | 0.066 | 0.096 | 2/3 |
| *EIF1AX/Y* | *OTTSUST00000007059* | OTTSUST00000013585 | 435 | 0.568 | 0.006 | 0.011 | 2/3 |
| *ZFX/Y* | *OTTSUST00000012891* | OTTSUST00000006293 | 2406 | 0.157 | 0.016 | 0.102 | 2/3 |
| *DDX3X/Y* | *OTTSUST00000012557* | OTTSUST00000012965 | 1989 | 0.478 | 0.020 | 0.042 | 2/3 |
| *AMELX/Y* | *OTTSUST00000001822* | OTTSUST00000012942 | 570 | 0.046 | 0.009 | 0.196 | 2/3 |
| *OFD1X/Y* | *OTTSUST00000001986* | OTTSUST00000013616 | 3003 | 0.090 | 0.039 | 0.433 | 2/3 |
| *USP9X/Y* | *OTTSUST00000008391* | OTTSUST00000006267 | 2556 | 0.417 | 0.032 | 0.077 | 2/3 |
| *EIF2S3X/Y* | *OTTSUST00000004949* | OTTSUST00000013587 | 472 | 0.355 | 0.007 | 0.020 | 2/3 |
| *TMSB4X/Y* | *OTTSUST00000005866* | OTTSUST00000013038 | 44 | 0.041 | 0.009 | 0.220 | 2/3 |
| *TXLNGX/Y* | *OTTSUST00000005375* | OTTSUST00000013706 | 473 | 1.194 | 0.129 | 0.180 | 2/3 |
| *TSPYL2/TSPY* | *OTTSUST00000004093* | OTTSUST00000013709 | 481 | 1.553 | 0.767 | 0.494 | 2/3 |

**Table S15** - synonymous substitution rates (K_S_) across Y gene coding regions. Accessions are provided for the transcripts aligned.

**2) XY-homology region around TRAPPC2-OFD1-GPM6B**

There is a large region (90.25kb) of high sequence identity (90%) between the X and Y chromosomes surrounding the genes *TRAPPC2-OFD1-GPMB6*. The region of homology runs from 10380557-10471811 on the Y assembly (90.25kb) and 11738536-11818708 (80.17kb) on the X assembly (Figure S1A).

**Supplementary figure 1 - OFD1 region (overleaf):**

OFD1Y transposed region compared to SSCX. A) Dotplot comparison of X and Y sequences. B) The homology region breaks down at a MER5B transposable element. C) Schematic view of the sequences. The breakpoint region is shown with a gradual degradation of sequence identity within the transposon. The transposed region presumably continues distally on the Y, but we do not have sequence here. C) A distinctive pattern of repetitive elements covering 5kb is found in two Y contigs with 99% sequence identity. This likely resulted from a transposition of sequence at the ERV elements at either end. The sequence appears to have originated near *KDM5D*; a LINE element within the intron of *OFD1Y* is disrupted by the insertion, with only the final 3kb remaining.


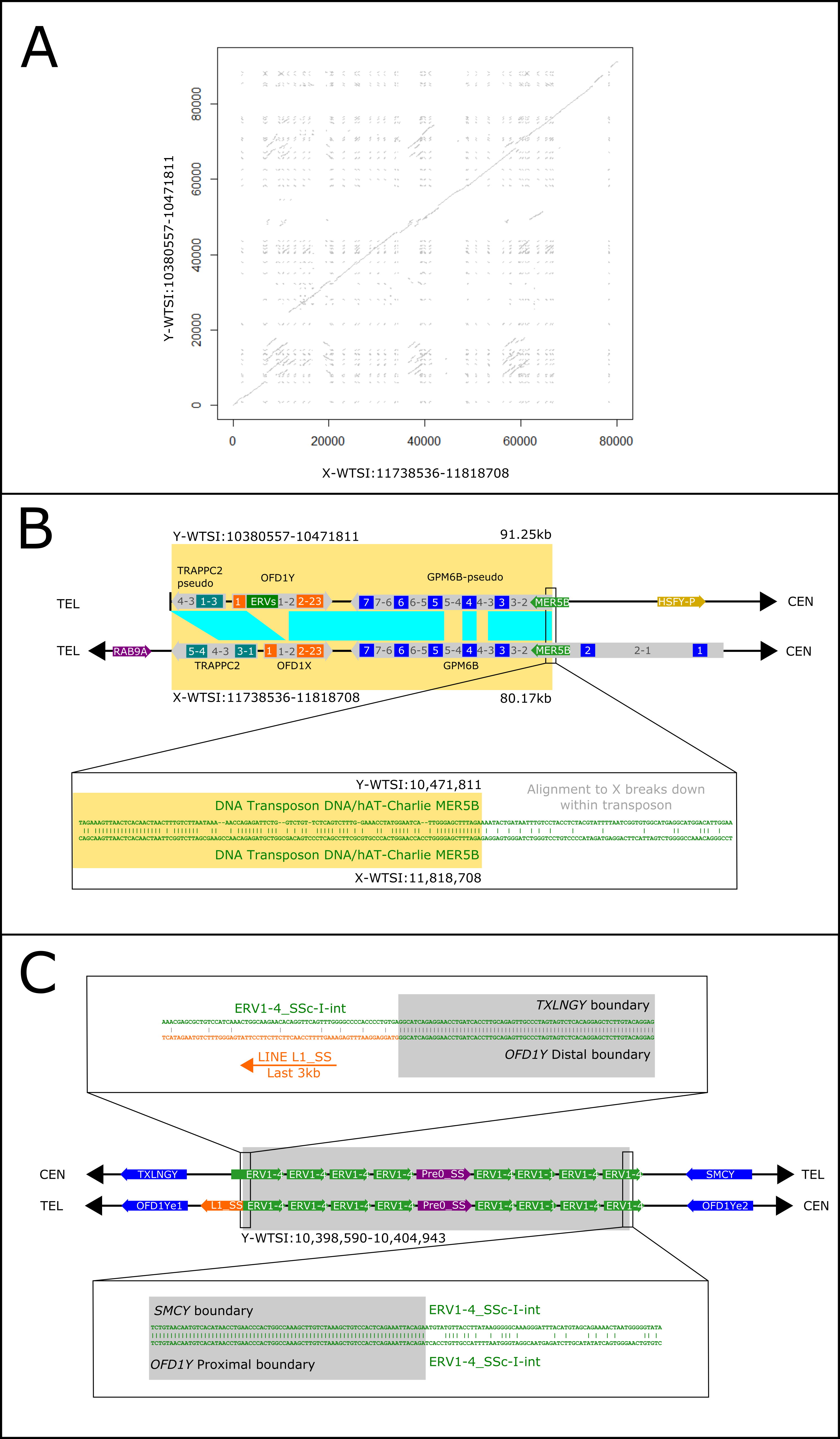


The boundary at the distal end is marked by the end of the associated Y contig, with a gap of ~120kb before the next distal contig. Consequently the homology region may extend distally by up to 120kb. The boundary lies in the intron between the third and fourth exons of *TRAPPC2* (OTTSUST00000013615). The three exons in the Y copy of *TRAPPC2* do not form an open reading frame, and thus *TRAPPC2* is a pseudogene on the Y regardless of the true homology block boundary position.

The boundary on the proximal end occurs within the intron between exon two and exon three of *GPM6B*. *GPM6B* is in a reverse orientation on both assemblies; exons one and two of *GPM6B* are thus not present on the Y, rendering this a pseudogene. The sequence alignment breaks down within a DNA transposon, MER5B (Figure S1B).

The high sequence identity is found not just amongst the exons of the three genes within this region; it extends to the introns and intergenic sequence as well; see table S17 for the base substitution rates across the region. The alignment is interrupted internally by a cluster of endogenous retroviral elements within the first intron of *OFD1Y*. This cluster appears to have arisen via a duplication from a similar cluster between *KDM5D* and *TXLNGY* (Figure S1C)); the clusters have a 99% sequence identity over a 5kb region, with the breakpoint regions clearly visible within one ERV element (ERV1-4_SSC-I-int) upon alignment. The breakpoint at the OFD1Y distal end disrupts an LINE element (L1_SS), a class annotated as active within the pig lineage, indicating the insertion occurred relatively recently.

There are two main possibilities to explain how this XY homologous region arose:

1. This is an ancestral feature of the Y chromosome. The sequence identity has been maintained by gene conversion, as is seen in rodent ZFX/Y (Hayashida et al. 1992) and pig AMELX/Y (Marais & Galtier 2003). The location of the genes argues in favour of this hypothesis. The order of genes on the X (and on the putative ancestral Y) is *AMELX-TMSB4X-TRAPPC2-OFD1X-GPM6B*: the same order seen on the Y. Two factors argue against a retained ancestral sequence: firstly, size of the region. The previously described case of gene conversion in ZFX/Y involved the coding regions, not the complete gene and intergenic sequence; AMELX/Y show strong gene conversion, but still have reduced sequence identity at the untranslated regions (UTRs). Secondly, the homology region ends within *GPM6B*; if the region were ancestral, the remnants of exons one and two should be present (unless a further chromosomal rearrangement carried them elsewhere. We have no evidence for *GPM6B* exons one or two in the available Y sequence).
2. The region has been recently transposed from the X. The OFD1Y gene has acquired new functionality in the testis, while the introns, intergenic regions and fragments of TRAPPC2 and GPM6B have begun diverging. The proximal breakpoint location within a transposable element argues in favour of this scenario by providing a mechanistic basis for a transposition event. Arguing against the idea, if this region has transposed, where are the X-degenerate copies of TRAPPC2, OFD1 and GPM6B? We did not find any similar sequences elsewhere on the Y; however, we have not assembled the entire single-copy region of the chromosome.

*OFD1Y* is an active gene in cattle (Chang et al. 2013). The published horse (*Equus caballus*) Y physical map (Raudsepp et al. 2004, Paria et al. 2011) does not contain *OFD1Y*. However, a draft sequence assembly reported at a recent Plant and Animal Genomes conference by Raudsepp et al suggests that *OFD1Y* is present and X-degenerate (slides available at https://pag.confex.com/pag/xxiii/recordingredirect.cgi/id/1824). Our own data show OFD1Y to be more highly expressed in testis than brain or kidney (Figure S2).


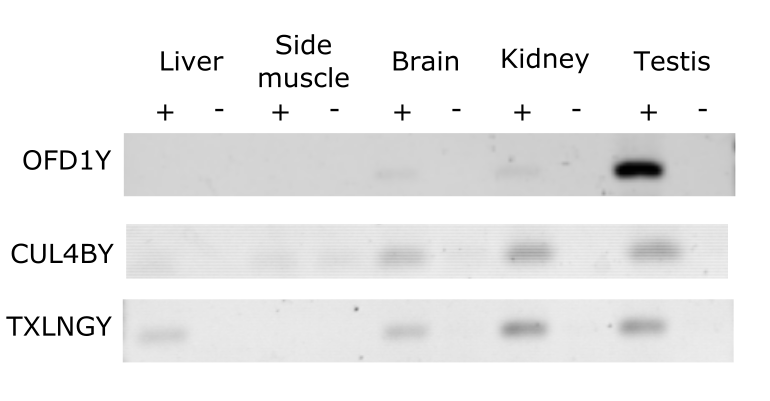


**Supplementary figure 2 – Gene expression**

RT-PCR shows strong expression of the X-transposed gene OFD1Y in testis, with lesser expression in brain and kidney. CUL4BY and TXLNGY have testis expression, plus expression in brain and kidney. Primers are given in supplementary table S14.

Cats lack *OFD1*, suggesting the gene was lost during carnivore evolution. However, Li et al (2011) have suggested that the similar ~120kb region *TRAPPC2-OFD1-GPM6B* has been subject to transposition onto the Y from the X chromosome in dogs. Other transposition events involving OFD1 are known. A transposition affecting the *RAB9A–SEDL–OFD1Y* genes has occurred in the human lineage (Chang et al 2011). Pseudogene copies of OFD1 in the autosomes of primates and rodents is thought to have occurred via retroposition of *OFD1X* (Chang et al, 2011).

Consequently, if the region in pig results from a transposition, there may have been a loss (or movement to an unsequenced part of the chromosome) of the X-degenerate copy, after the divergence with cattle (~63 million years ago), followed by a transposition into a region restoring the ancestral relationship to AMELX/Y and TMSB4X/Y, perhaps guided by existing homologies. Based on our reconstruction of a pathway from the ancestral Y chromosome to the pig Y (supplementary figure 12), we would expect the ancestral *OFD1Y* to lie between *AMELY* and the HSFY block.

We believe a transposition is a plausible explanation for these observations. As further sequence is generated, this should resolve. If the distal boundary of the homology region lies within a transposable element, and if sequences resembling X-degenerate copies of the three genes involved are found elsewhere on the chromosome, there will be strong evidence for a transposition event.

| Region | Aligned Size (bp) | Base substitutions per site (standard error estimate) |  |
| --- | --- | --- | --- |
| TRAPPC2 introns | 536 | 0.009 (0.004) |  |
| TRAPPC2 exons | 91 | 0.022 (0.016) |  |
| OFD1 exons | 3003 | 0.050 (0.004) |  |
| OFD1 introns | 60898 | 0.256 (0.002) |  |
| OFD1-GPM6B intergenic region | 15386 | 0.165 (0.004) |  |
| GPM6B introns | 9848 | 0.302 (0.006 ) |  |
| GPM6B exons | 630 | 0.063 (0.010) |  |

**Table S16: Sequence divergence between X and Y copies of the potentially transposed region around *TRAPPC2-OFD1-GPM6B* (Y-WTSI:10380557-10471811 and X-WTSI:11738536-11818708).**

**3) Differences between pig X and human X**

1. **Loss Of Function genes on the pig X**

Eleven protein coding genes present on the human X have been annotated as unitary pseudogenes (also known as Loss Of Function genes) on the pig X (Supplementary Table S10). Genes with potential biological relevance include:

*GUCY2F* (OTTSUSG00000005153): in humans this gene has been suggested as a possible candidate for involvement in X-linked retinitis pigmentosa (RP) (Yang et al. 1996). Pig models are now being developed for studying RP, taking advantage of the similarities between human and porcine retinal development (e.g. Ross et al. 2012; Fernandez de Castro et al. 2014). These models benefit from an improved understanding of the status of orthologues of disease-related genes.

*AWAT1* (OTTSUSG00000002936): this gene is found on the X chromosome in a wide range of mammals, including cattle, humans and opossums (Holmes 2010). It encodes an acyl CoA wax alcohol acyltransferase involved in sebum production, and in humans is expressed in sebocytes, aiding in the prevention of surface desiccation of the skin (Turkish et al. 2005). The gene is a member of a family including *DGAT1* and *DGAT2*, which have previously been associated with backfat thickness and intramuscular fat content respectively in pig breeds (Cui et al. 2011). It is likely that this pseudogenised *AWAT1* in Duroc pigs represents a species-wide trait, as no transcripts for *AWAT1* appear in the EST databases from any pig breeds.

*ITIH6* (OTTSUSG00000005361): a trypsin inhibitor that interacts with the extracellular matrix, known to be conserved in chimpanzee, Rhesus monkey, mouse, chicken, zebrafish, and frog.

*RAB41* (OTTSUSG00000005397): a member of the RAS oncogene family, as well as a number of zinc finger proteins and a transmembrane protein. This gene is active in primates, as well as in dolphin, hedgehog and megabat; the loss likely represents a pig-lineage specific event.

**2) Other regions of interest**

Other regions of difference lie in the cancer/testis (*CT*) antigen clusters found in humans and other primates but which are significantly reduced in pig. This is in line with evidence that enlarged primate CT antigen clusters arose due to a recent amplification in primates (Zhang and Su 2014), perhaps driven by a retrotransposition event. Their potential functions remain unknown, though they may have been involved in primate speciation. Some members of the CT family of genes have also expanded in other lineages - for example the SSX family on the mouse X chromosome, which contains 13 genes with a similar expression pattern to human: testis and tumors (Chen et al., 2003).

*INE1* (Inactivation-escape-1) lacks evidence in pig. This is a non-coding transcript with unknown function within an intron of *UBE1X* (Thiselton et al. 2002). It appears to be only be conserved in primates, where it escapes X inactivation in females.


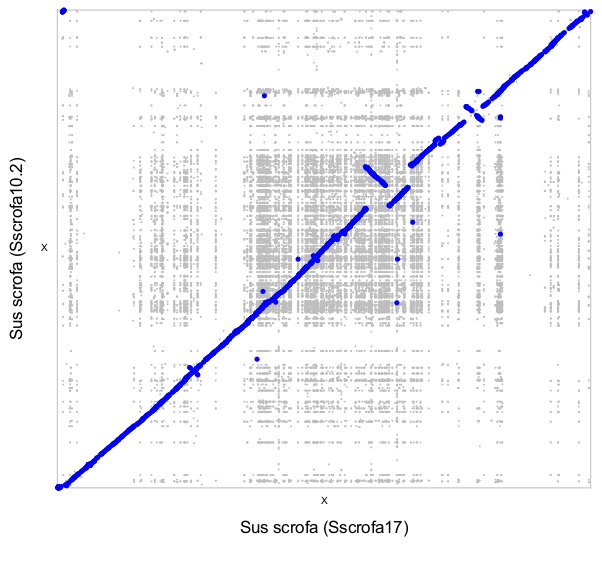


**Supplementary figure 3 - 10.2 X vs this X-WTSI**

Dot plot between the old and the new pig X sequence assemblies. Low-sensitivity alignments between both versions are shown in grey. A more stringent alignment is shown with thick blue lines and highlights large-scale differences between both versions.


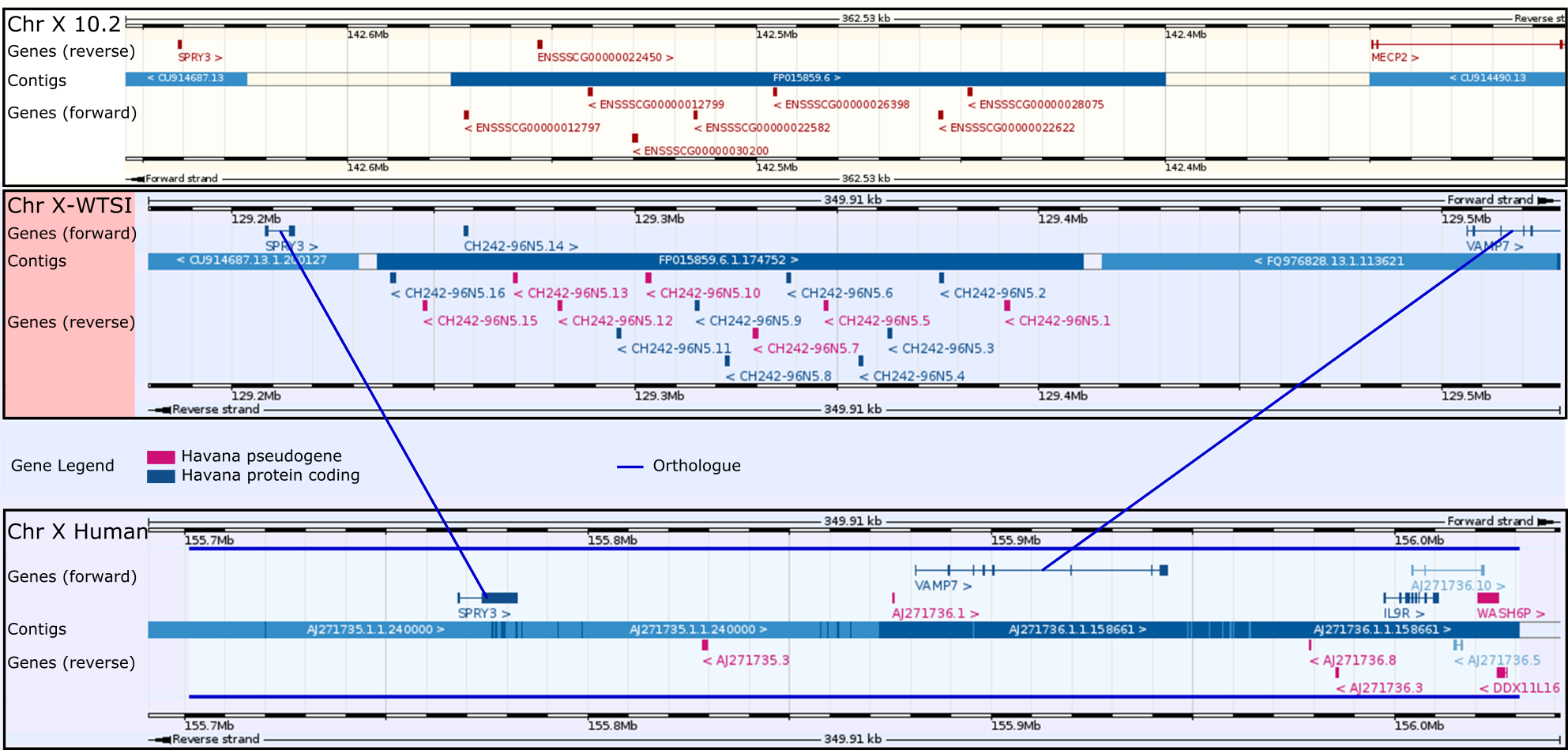


**Supplementary figure 4 – Assembly improvement around an X-chromosome**

**olfactory receptor gene cluster expansion**

Pigs have an expansion of olfactory receptors throughout the genome; two clusters lie on the pig X, not found in humans. The region provides an example of the sequence improvement from optical mapping between the 10.2 assembly (top; image from Ensembl) and the current assembly (centre), with a comparison to the corresponding region on the human X (bottom; both images from Vega). The improvement in the current assembly allowed reorientation (Ensembl image has been flipped for clarity), the correct positioning of the VAMP7 gene which was previously annotated on an unplaced scaffold and improved annotation of the genes (blue) and pseudogenes (pink) within the region.


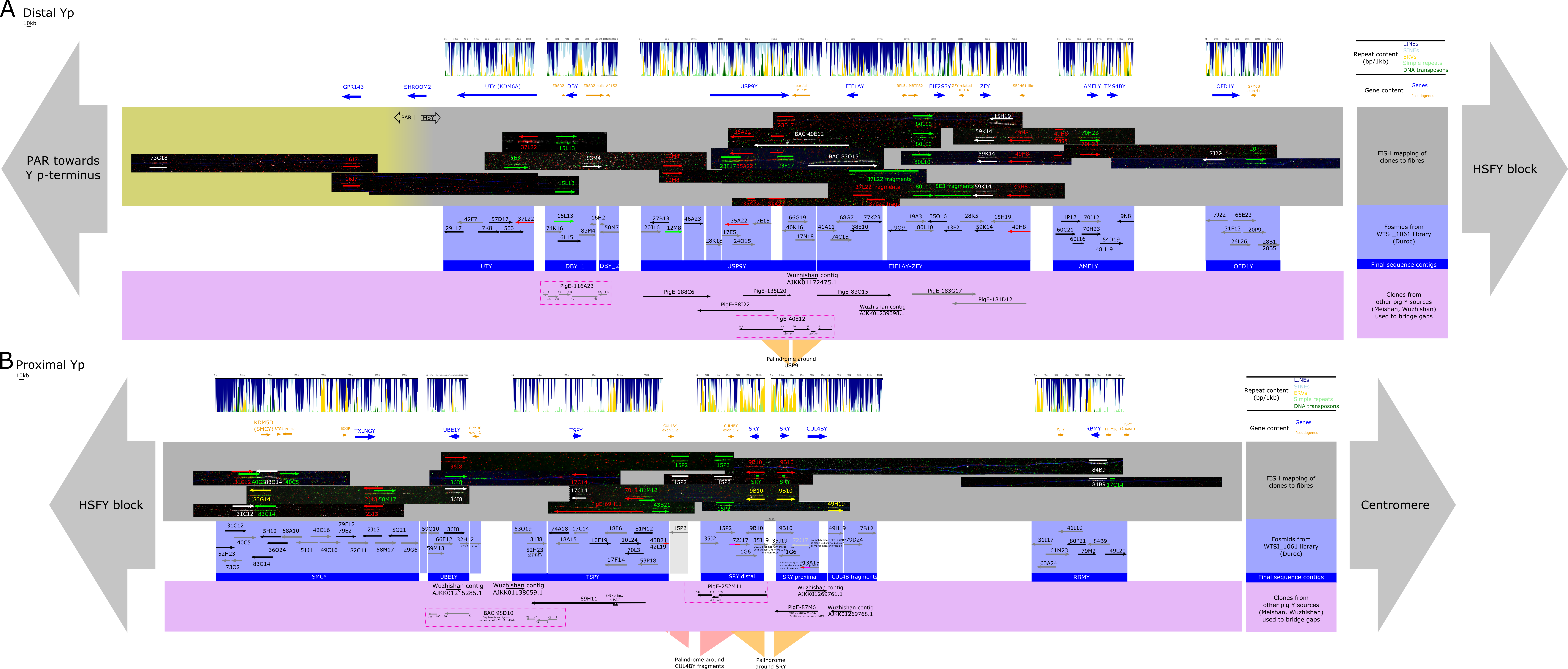


**Supplementary figure 5 - Detailed display of contigs**

The figure shows A) the distal and B) the proximal blocks of genes on the Y short arm. The light blue backgrounded region shows the overlapping fosmids from the Duroc Y library WTSI_1061. These sequences were contiged, and gaps were filled using Y sequences originating from other pig breeds (PigE BAC clones from Meishan, and scaffold sequences from the Wuzhishan sequencing project (Fang et al. 2012). The final sequence contigs obtained are shown by the dark blue panels. The contigs were ordered and anchored to the chromosome by FISH mapping of clones on DNA fibres; the fibre images are tiled in the grey panels. Gene and pseudogene positions are annotated above the fibres, as well as the repetitive density within 1kb windows along each final contig sequence obtained via RepeatMasker. Three palindromic regions are seen: firstly in the distal block around *USP9Y*; secondly around the *CUL4B* fragments, and finally around the *SRY* copies in the proximal block.


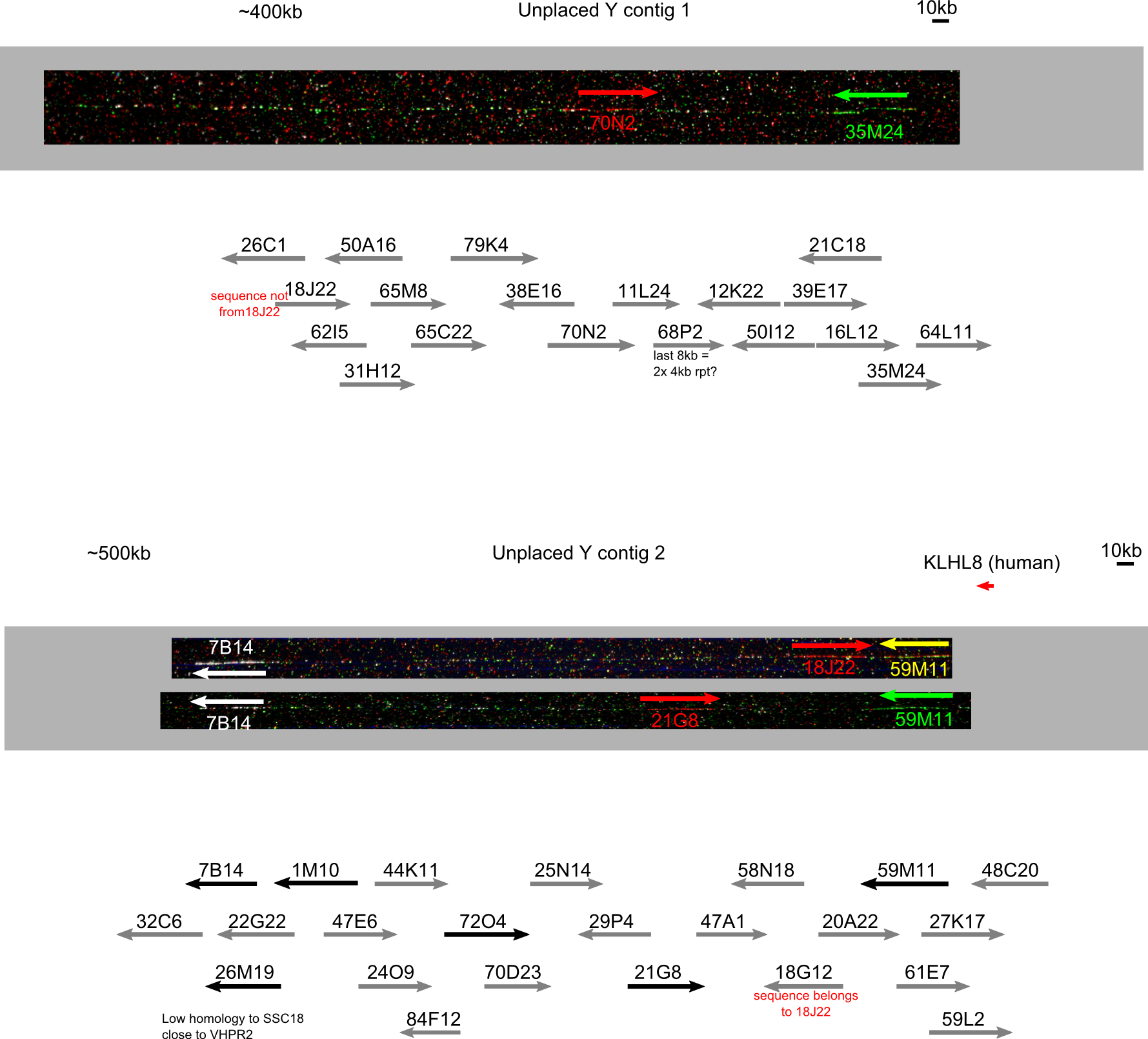


**Supplementary figure 6 - large unplaced contigs**

Unplaced Y contigs. Fibre FISH could not link these to any other sequence contigs. Metaphase FISH places them on Yp, close to the centromere (fig S4). The clones 18J22 and 18G12 have swapped identifiers i.e. the database sequence with the identifier 18G12 originated from clone 18J22, explaining the unexpected hybridisation pattern.


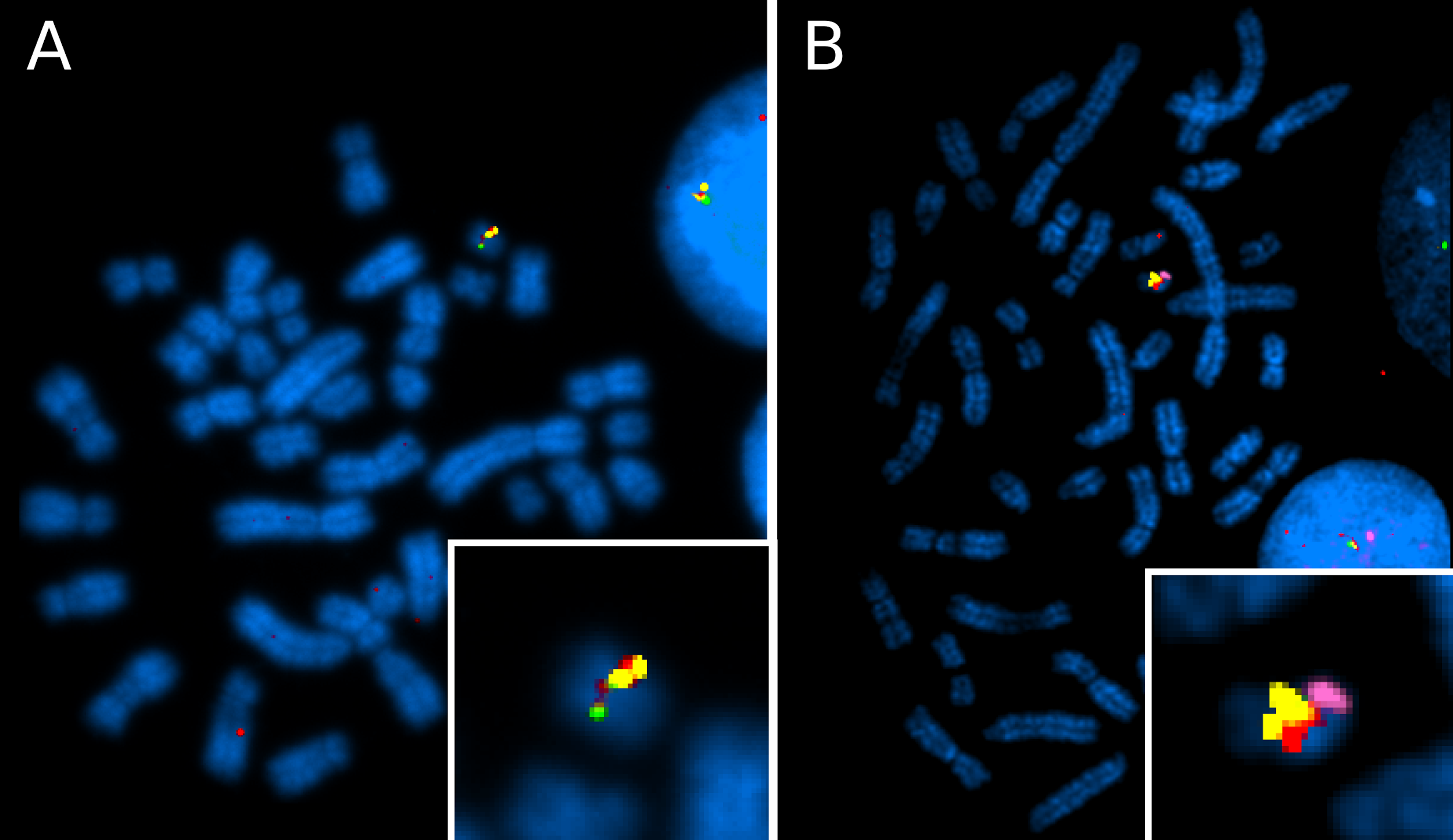


**Supplementary figure 7 - unplaced contigs are near the centromere of Yp**

Metaphase FISH using WTSI_1061 library fosmid clones from the unplaced contigs shown in figure S3. The Y chromosome is expanded in the inset.

A) Unplaced contig 1: Red: 70N2; yellow: 18J22; green: 35M24

B) Unplaced contig 2: Red: 21G8; yellow: 7B14; green: 59M11: purple: USP9Y-containing 12M8, to show chromosome orientation..

A)


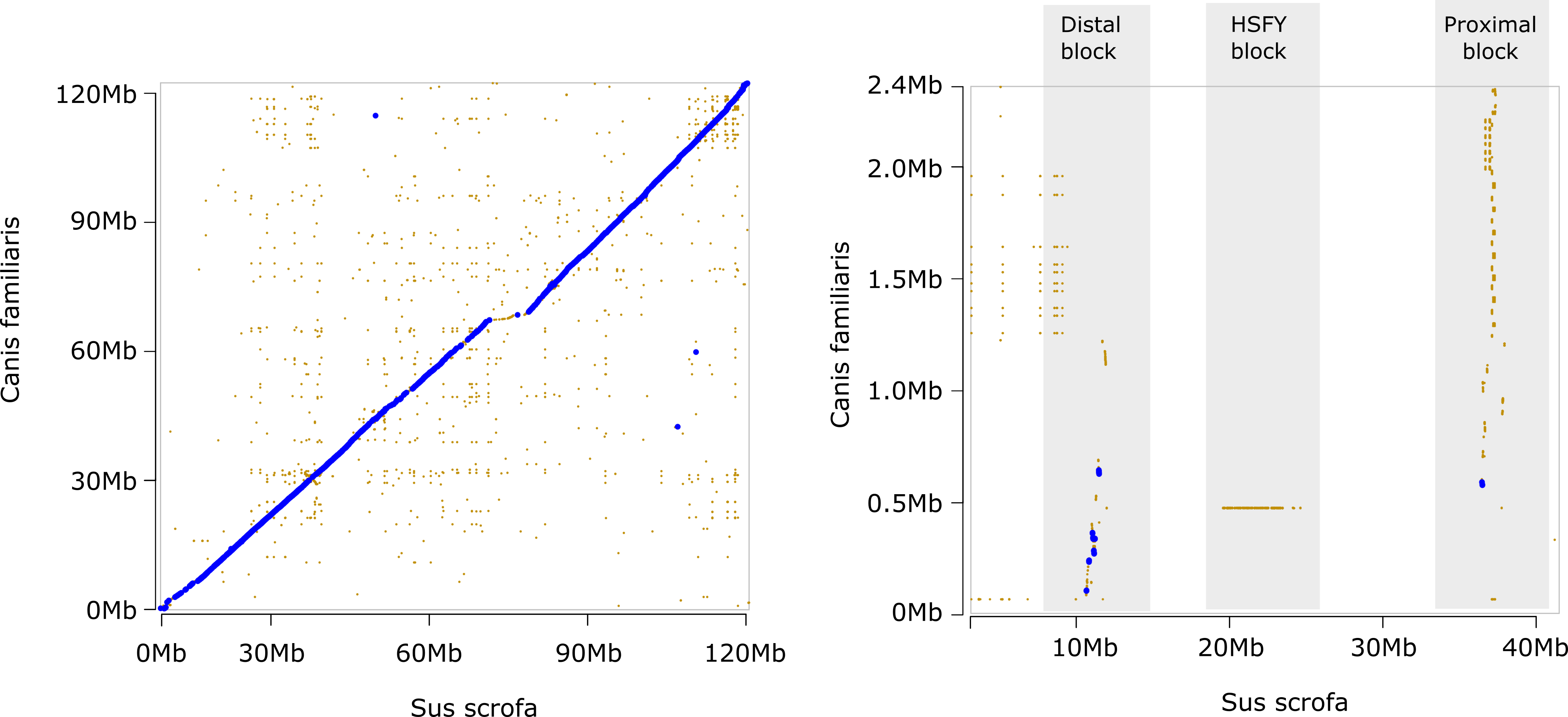


B)


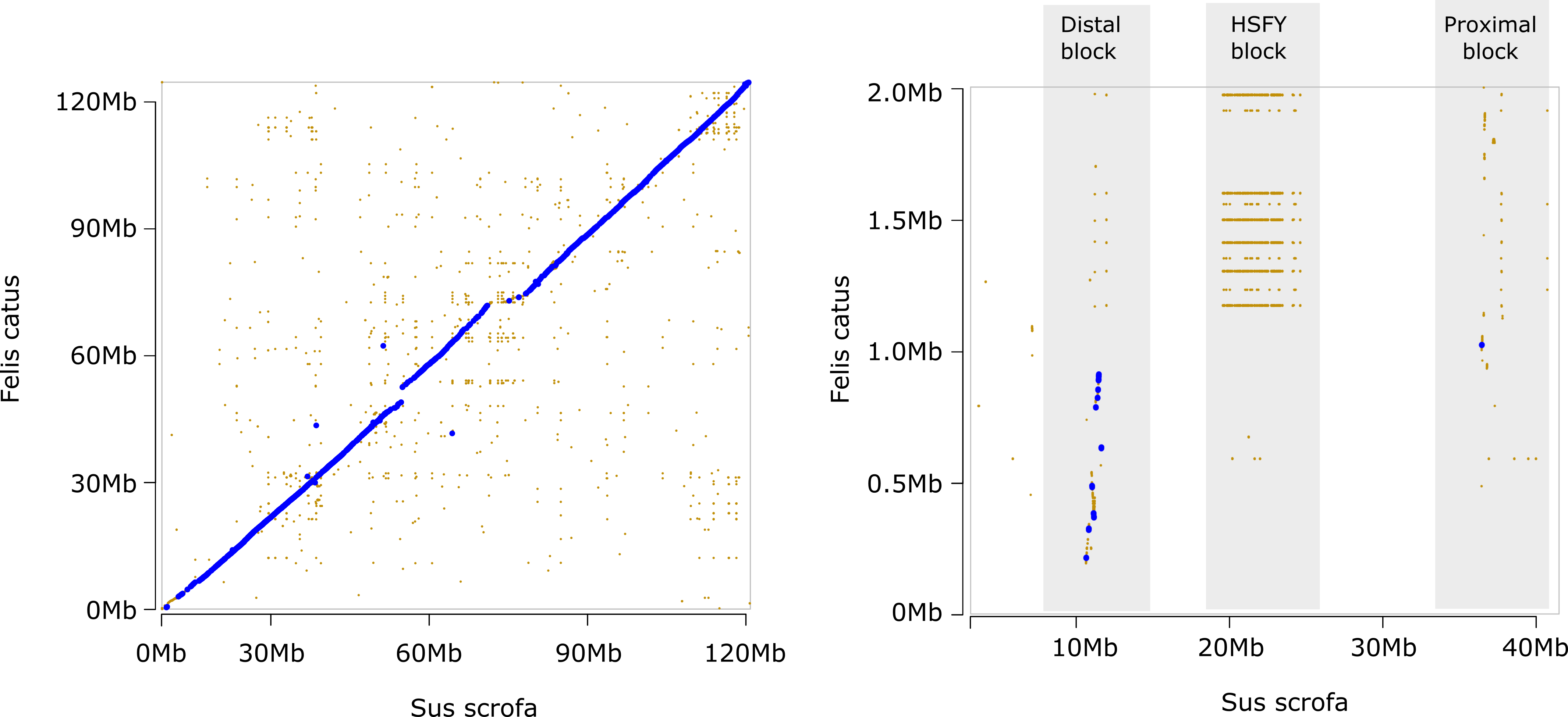


C)


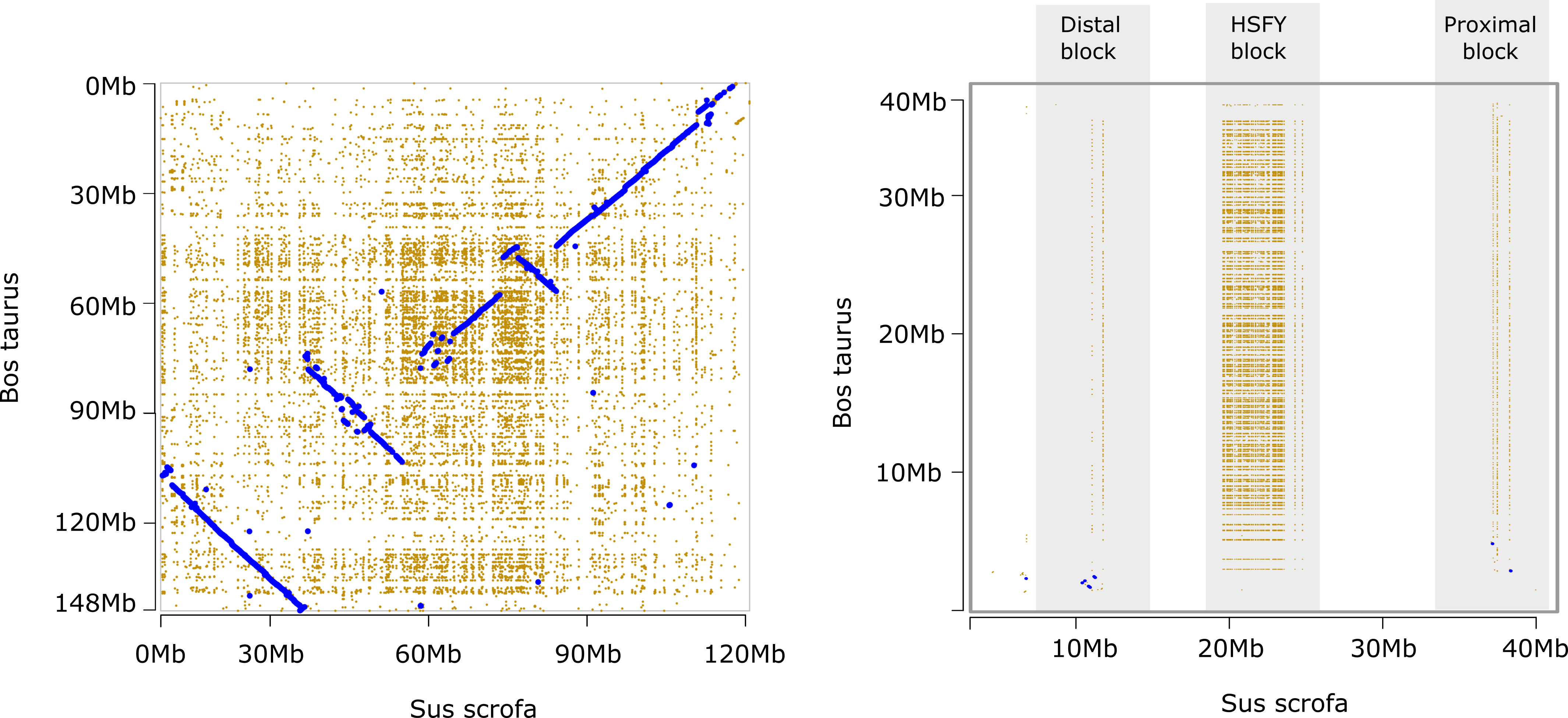


D)


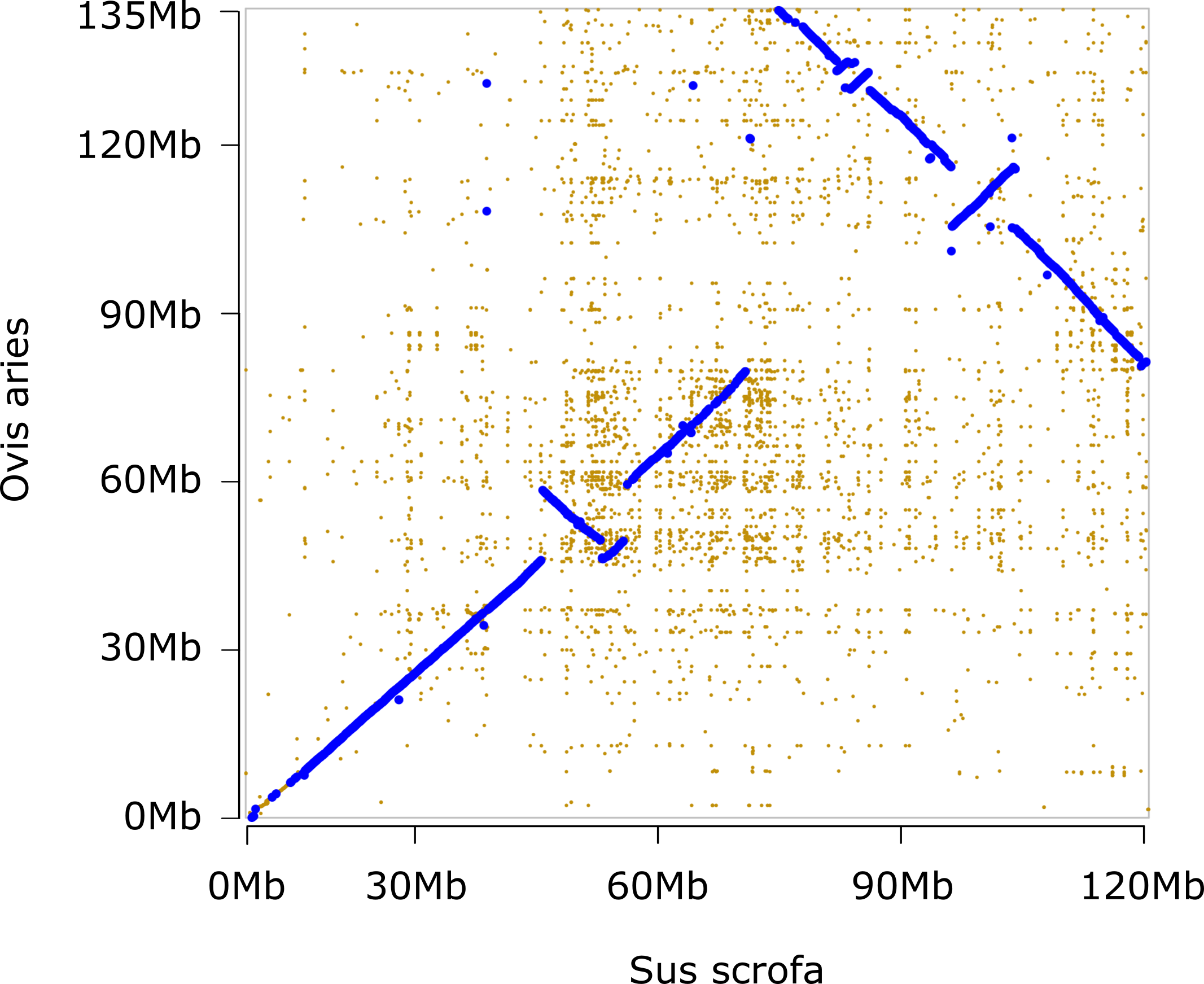


E)


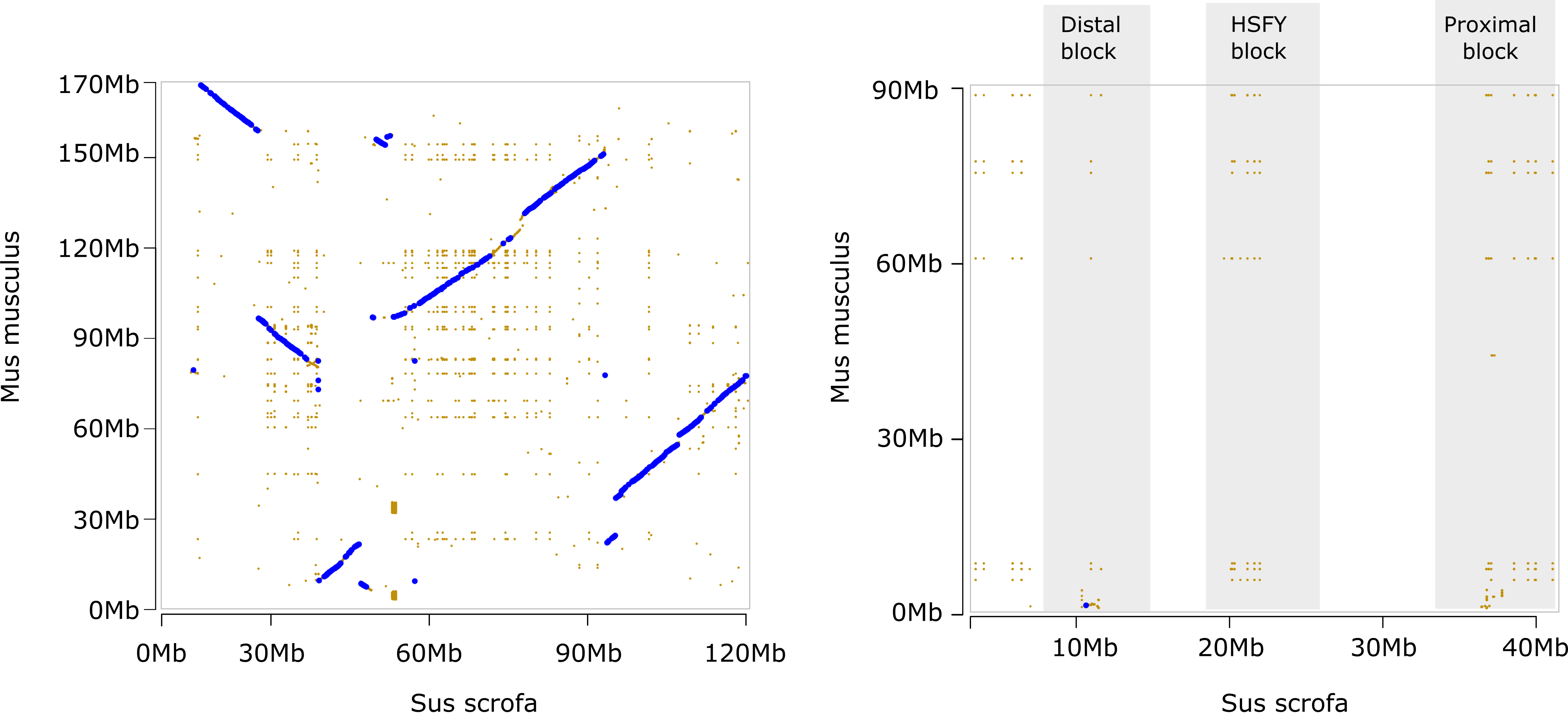


F)


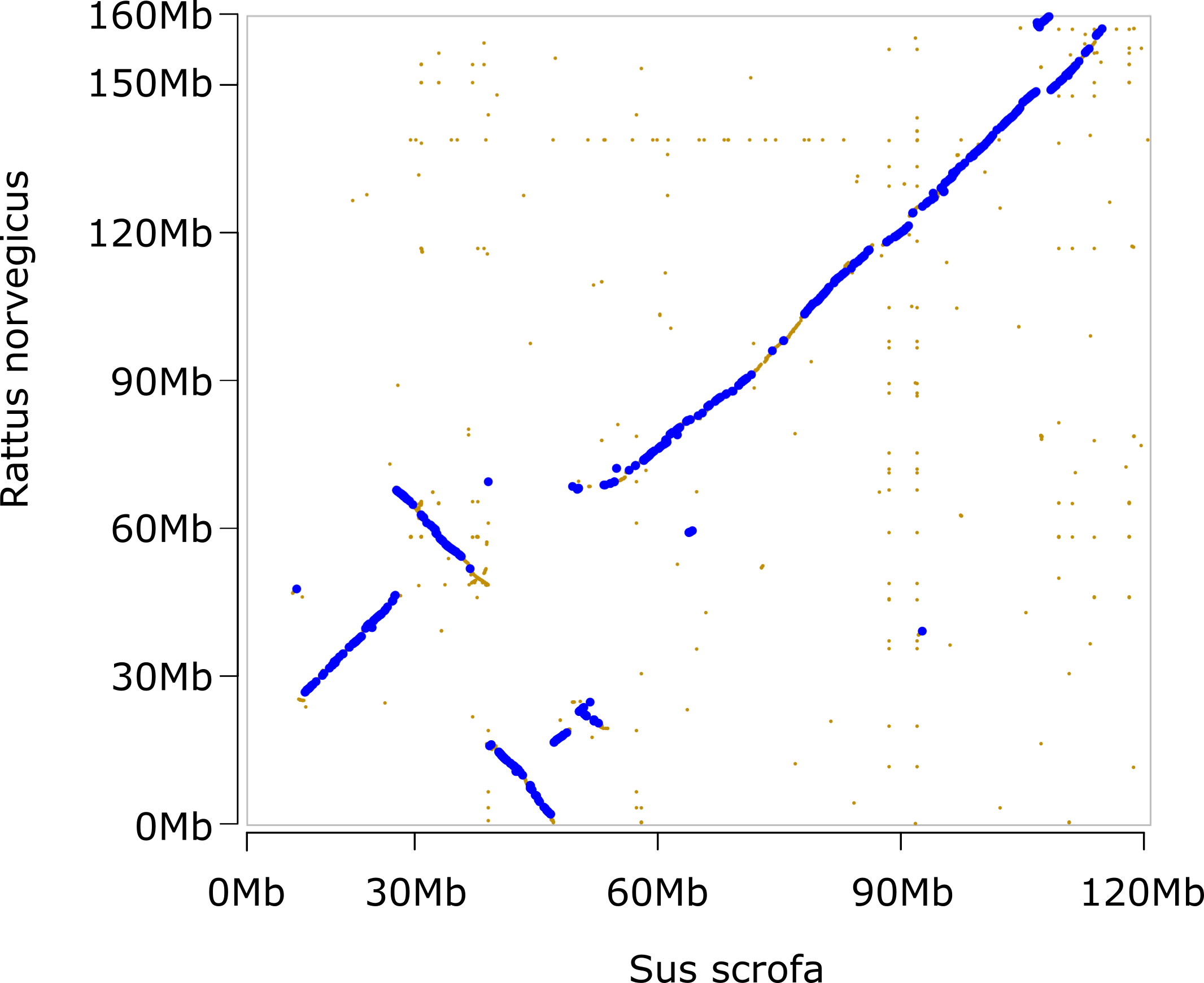


G)


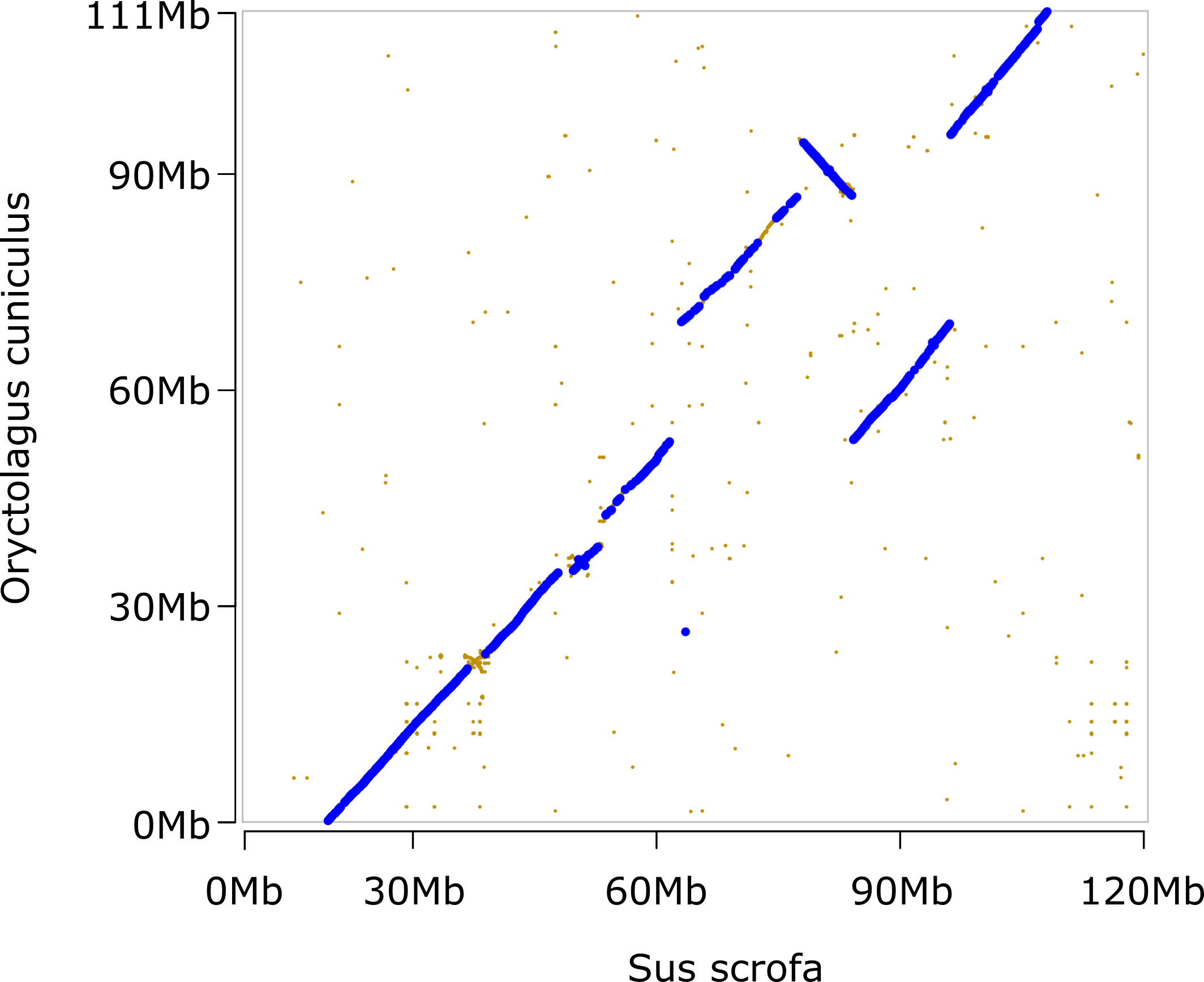


H)


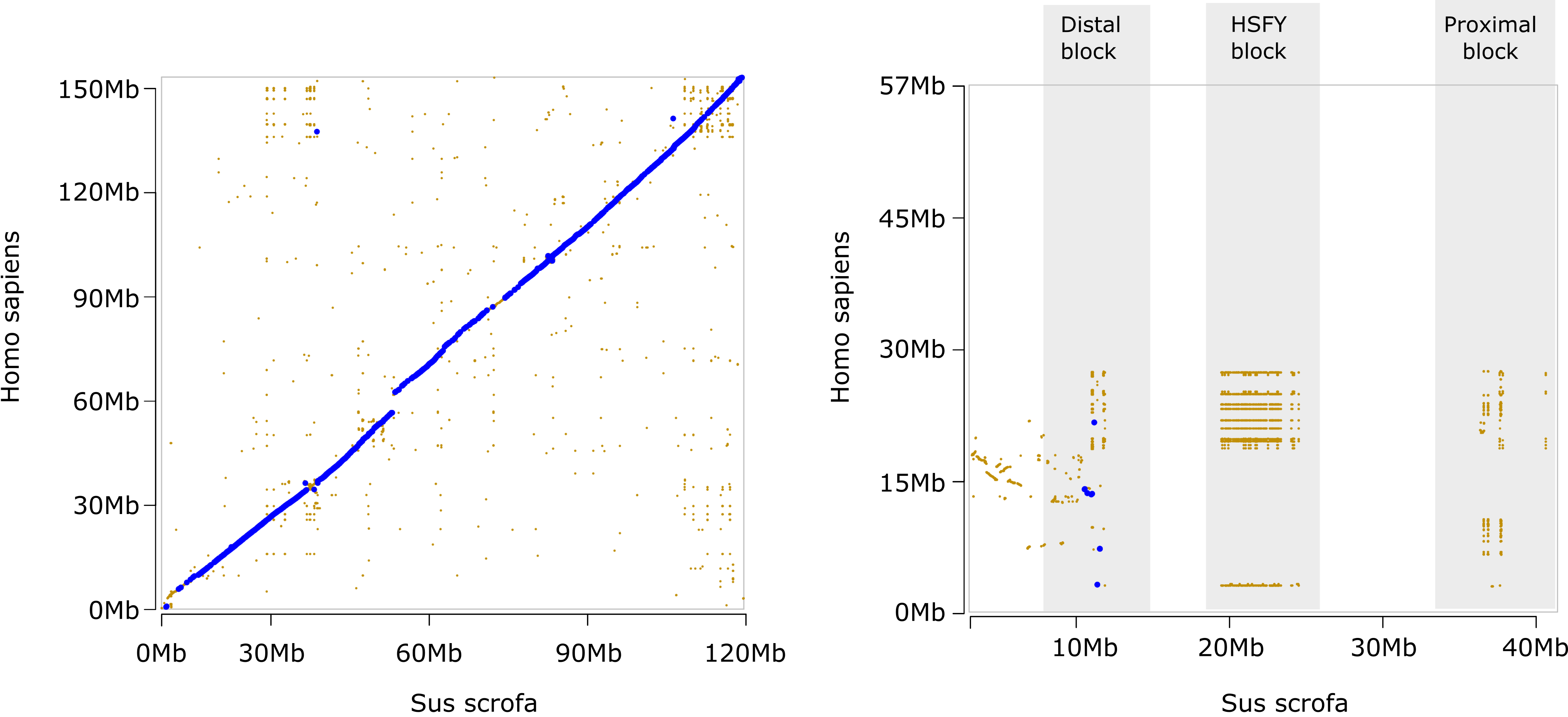


I)


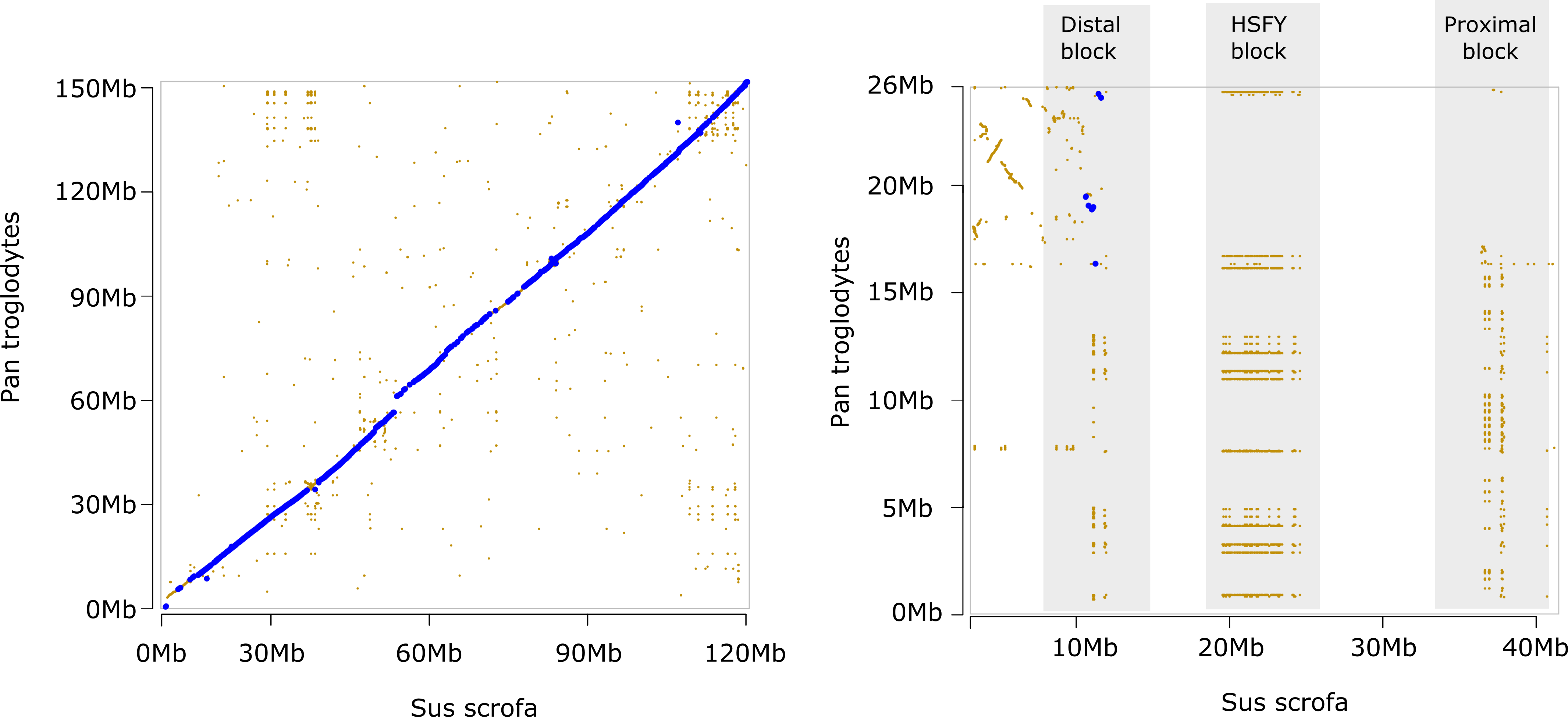


**Supplementary figure 8 - cross-species comparisons between X and Y chromosomes**

Expanded versions of the alignments shown in Figure 1. The X chromosome is in the left, and the Y (where available) is on the right. A) Dog B) Cat C) Cow D) Sheep E) Mouse F) Rat G) Rabbit H) Human I) Chimp.

The position of the gene clusters in the pig Y are indicated; species-specific gene expansions can be seen, for example the multiple SRY copies in dogs (proximal block), and the large HSFY expansion in cattle (HSFY block).


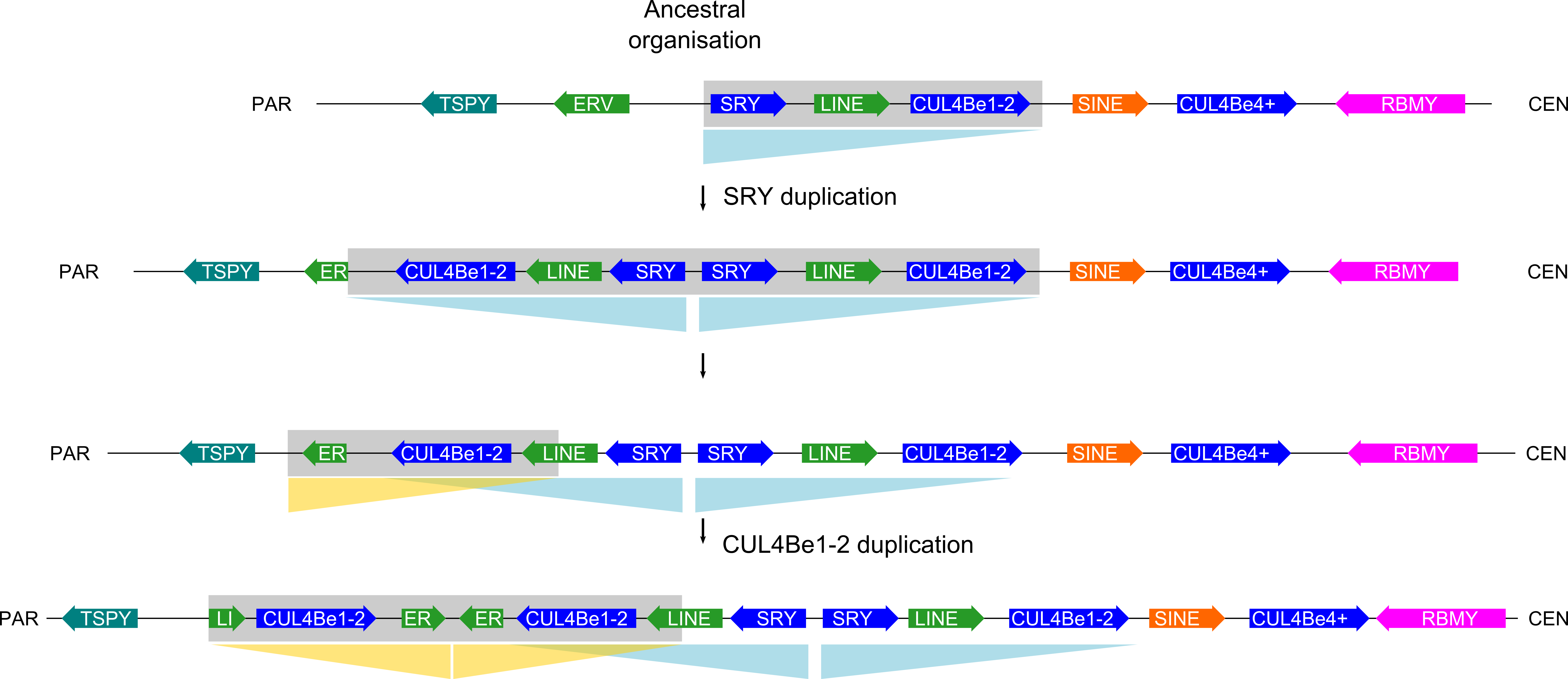


**Supplementary figure 9 - *SRY* and *CUL4B* order of inversions**

Scenario showing duplications of sequence abound *SRY* and *CUL4B* from an ancestral state of single copy *SRY* and *CUL4B*. The breakpoint regions are detailed in Figure 4. Resulting palindrome arms are highlighted.


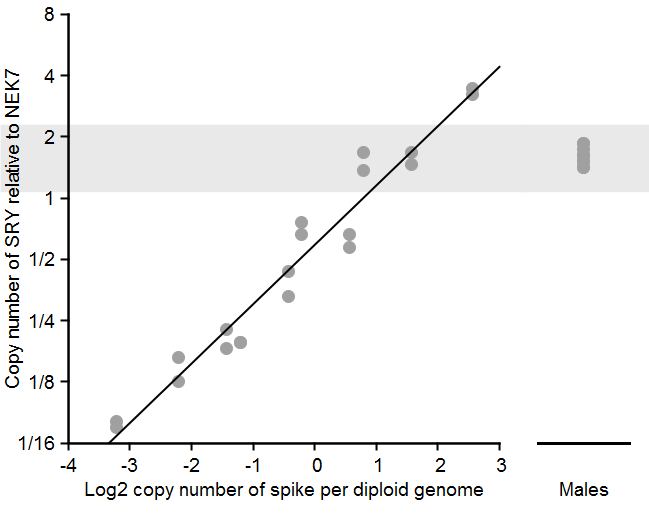


**Supplementary figure 10 - qPCR of SRY copy number**

The copy number of *SRY* in S. scrofa was estimated. Female gDNA was spiked with known concentrations of the *SRY* PCR product (supplementary table 6) and copy number relative to the single copy control gene *NEK7* was used to construct a standard curve. The same measurement was made with gDNA from four male pigs. The results are consistent with an *SRY* copy number greater than one, but there are unlikely to be more than two copies in the genome.


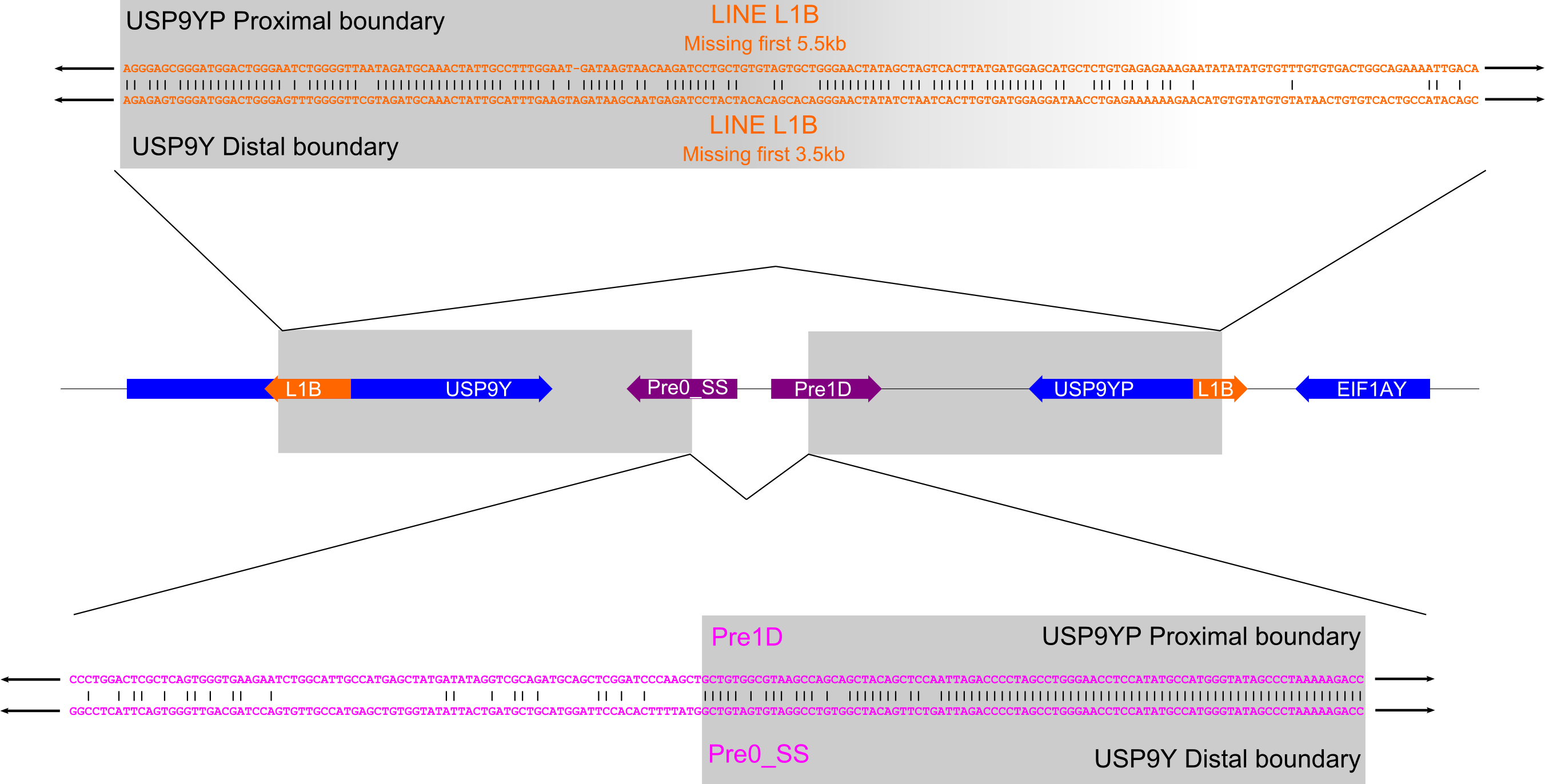


**Supplementary figure 11 - *USP9Y* breakpoints**

The breakpoint regions either side of the USP9Y inversion are shown in detail. Both boundaries encompass transposable elements. The degradation of sequence identity is more gradual than in the *SRY* and *CUL4B* duplications, taking place over some tens of base pairs, suggesting this was an earlier event.


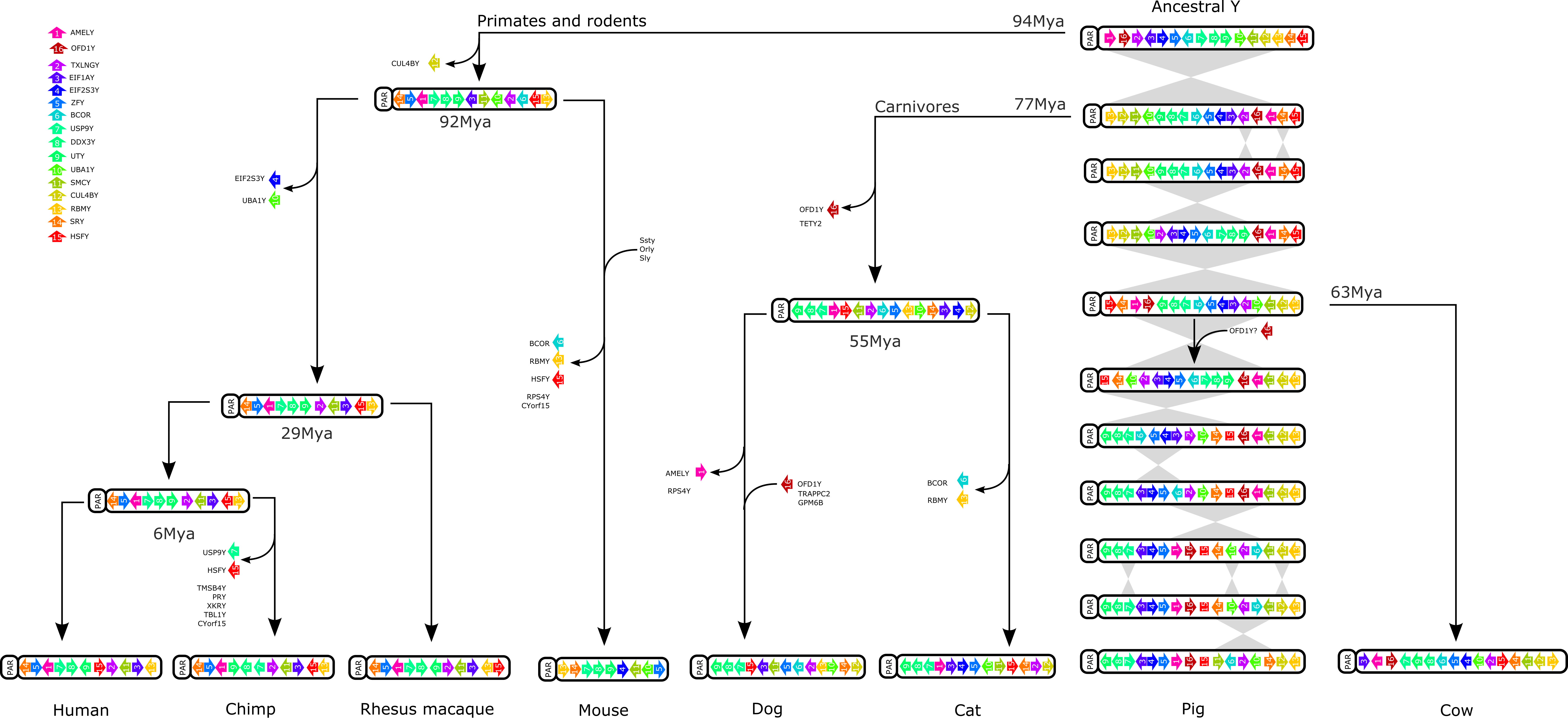


**Supplementary Figure 12 – evolutionary rearrangements on the Y chromosome**

Previous reconstructions of ancestral Y chromosomal organisations based on gene order (Li et al. 2013a) were combined with our pig data and existing cattle data (Elsik et al. 2009). Optimal ancestral states and pathways were constructed using MGR. The rearrangements required to transform from the putative ancestral Y chromosome to the pig Y are shown. . Gains and losses of other genes are shown as described in Li et al (2013a). Only one group of genes remain intact across all studied mammals here as a syntenic block - *KDM6A-DDX3Y-USP9Y* (light blue shading). This is consistent with the suggestion that these genes have a functional requirement to remain in proximity. The potential *OFD1* region transposition from the X is noted after the divergence with cattle; the rearrangement pathway suggests that if the ancestral *OFD1Y* sequence has been retained, it will be found between *AMELY* and *HSFY*.


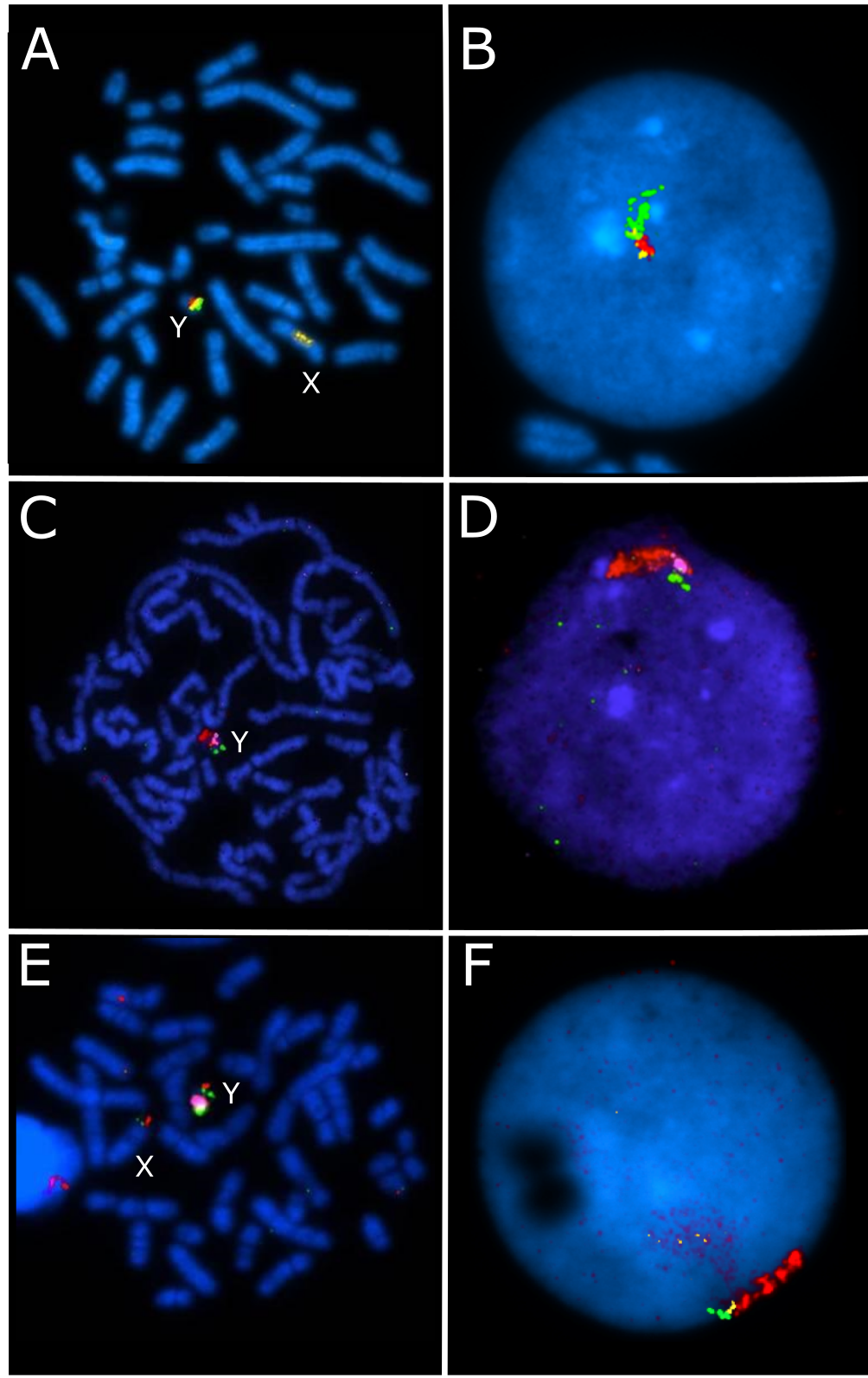


**Supplementary figure 13 – FISH results**

*Amplification of sequences on proximal Yp*

FISH with WTSI_1061 library fosmid clones containing *UBE1Y* (36I8 -yellow), *TXLNGY* (2J13 – red), *KDM5D* (58M17 – green) in A) metaphase and B) interphase. All clones are on Yp, but sequences within 58M17 have amplified towards the centromere. Hybridisation of sex chromosome enriches repetitive content is also visible on Xq.

*Amplification of sequences on Yq*

FISH on C) metaphase and D) interphase nucleus with clones containing *TSPY* (17C14 – red), *KDM5D* (40C5 - purple) and *DDX3Y* (15L13 – green) showing the expansion of 17C14 related material along the distal region of Yq.

*Low copy region at Yq terminus*

E) FISH on metaphase with clones 73G18 (purple - much of Yq), 74D22 (red - PAR) and 23F17 (green - PAR, Yp at USP9Y, and Yq terminus), demonstrating that some lower copy number sequences are present at the Yq terminus.

F) FISH on interphase with CUL4B-containing clones 15P2 (green), 49H19 (yellow) and 13A5 (red), demonstrating expansion of material in 13A5 proximal to SRY.

**Supplementary References**

Bonfield JK, Smith K f, Staden R. 1995. A new DNA sequence assembly program. *Nucleic Acids Res* **23**: 4992–4999.

Bonfield JK, Whitwham A. 2010. Gap5--editing the billion fragment sequence assembly. *Bioinforma Oxf Engl* **26**: 1699–1703.

Bourque G, Pevzner PA. 2002. Genome-scale evolution: reconstructing gene orders in the ancestral species. *Genome Res* **12**: 26–36.

Chain PSG, Grafham DV, Fulton RS, Fitzgerald MG, Hostetler J, Muzny D, Ali J, Birren B, Bruce DC, Buhay C, et al. 2009. Genomics. Genome project standards in a new era of sequencing. *Science* **326**: 236–237.

Dawson HD, Loveland JE, Pascal G, Gilbert JGR, Uenishi H, Mann KM, Sang Y, Zhang J, Carvalho-Silva D, Hunt T, et al. 2013. Structural and functional annotation of the porcine immunome. *BMC Genomics* **14**: 332.

Dear S, Durbin R, Hillier L, Marth G, Thierry-Mieg J, Mott R. 1998. Sequence assembly with CAFTOOLS. *Genome Res* **8**: 260–267.

Elsik CG, Tellam RL, Worley KC. 2009. The Genome Sequence of Taurine Cattle: A Window to Ruminant Biology and Evolution. *Science* **324**: 522–528.

Gribble SM, Fiegler H, Burford DC, Prigmore E, Yang F, Carr P, Ng BL, Sun T, Kamberov ES, Makarov VL, et al. 2004. Applications of combined DNA microarray and chromosome sorting technologies. *Chromosome Res Int J Mol Supramol Evol Asp Chromosome Biol* **12**: 35–43.

Harris R. 2007. Improved pairwise alignment of genomic DNA. The Pennsylvania State University.

Harrow J, Frankish A, Gonzalez JM, Tapanari E, Diekhans M, Kokocinski F, Aken BL, Barrell D, Zadissa A, Searle S, et al. 2012. GENCODE: the reference human genome annotation for The ENCODE Project. *Genome Res* **22**: 1760–1774.

Humphray SJ, Scott CE, Clark R, Marron B et al. 2007. A high utility integrated map of the pig genome. *Genome Biology* **8**: R139.

Li G, Davis BW, Raudsepp T, Wilkerson AJP, Mason VC, Ferguson-Smith M, O’Brien PC, Waters PD, Murphy WJ. 2013a. Comparative analysis of mammalian Y chromosomes illuminates ancestral structure and lineage-specific evolution. *Genome Res* **23**: 1486–1495.

Li M, Tian S, Jin L, Zhou G, Li Y, Zhang Y, Wang T, Yeung CKL, Chen L, Ma J, et al. 2013b. Genomic analyses identify distinct patterns of selection in domesticated pigs and Tibetan wild boars. *Nat Genet* **45**: 1431–1438.

Loveland JE, Gilbert JGR, Griffiths E, Harrow JL. 2012. Community gene annotation in practice. *Database J Biol Databases Curation* **2012**: bas009.

Michalet X, Ekong R, Fougerousse F, Rousseaux S, Schurra C, Hornigold N, Slegtenhorst M van, Wolfe J, Povey S, Beckmann JS, et al. 1997. Dynamic Molecular Combing: Stretching the Whole Human Genome for High-Resolution Studies. *Science* **277**: 1518–1523.

Nei M, Gojobori T. 1986. Simple methods for estimating the numbers of synonymous and nonsynonymous nucleotide substitutions. *Mol Biol Evol* **3**: 418–426.

Ng BL, Carter NP. 2006. Factors affecting flow karyotype resolution. *Cytom Part J Int Soc Anal Cytol* **69**: 1028–1036.

Otto TD, Sanders M, Berriman M, Newbold C. 2010. Iterative Correction of Reference Nucleotides (iCORN) using second generation sequencing technology. *Bioinforma Oxf Engl* **26**: 1704–1707.

Quail MA, Matthews L, Sims S, Lloyd C, Beasley H, Baxter SW. 2011. Genomic libraries: II. Subcloning, sequencing, and assembling large-insert genomic DNA clones. *Methods Mol Biol Clifton NJ* **772**: 59–81.

Quail MA, Swerdlow H, Turner DJ. 2009. Improved protocols for the illumina genome analyzer sequencing system. *Curr Protoc Hum Genet Editor Board Jonathan Haines Al* **Chapter 18**: Unit 18.2.

Seal RL, Gordon SM, Lush MJ, Wright MW, Bruford EA. 2011. genenames.org: the HGNC resources in 2011. *Nucleic Acids Res* **39**: D514–519.

Searle SMJ, Gilbert J, Iyer V, Clamp M. 2004. The otter annotation system. *Genome Res* **14**: 963–970.

Simpson JT, Wong K, Jackman SD, Schein JE, Jones SJM, Birol I. 2009. ABySS: a parallel assembler for short read sequence data. *Genome Res* **19**: 1117–1123.

Skinner BM, Griffin DK. 2012. Intrachromosomal rearrangements in avian genome evolution: evidence for regions prone to breakpoints. *Heredity* **108**: 37–41.

Skinner BM, Lachani K, Sargent CA, Affara NA. 2013. Regions of XY homology in the pig X chromosome and the boundary of the pseudoautosomal region. *BMC Genet* **14**: 3.

Smit A, Hubley R, Green P. 1996. *RepeatMasker Open-3.0*. http://www.repeatmasker.org.

Tamura K, Peterson D, Peterson N, Stecher G, Nei M, Kumar S. 2011. MEGA5: Molecular Evolutionary Genetics Analysis Using Maximum Likelihood, Evolutionary Distance, and Maximum Parsimony Methods. *Mol Biol Evol* **28**: 2731–2739.

Tsai IJ, Otto TD, Berriman M. 2010. Improving draft assemblies by iterative mapping and assembly of short reads to eliminate gaps. *Genome Biol* **11**: R41.

Zerbino DR. 2010. Using the Velvet de novo assembler for short-read sequencing technologies. *Curr Protoc Bioinforma Ed Board Andreas Baxevanis Al* **Chapter 11**: Unit 11.5.

#

# **Acknowledgements**

We wish to thank the following people in the Wellcome Trust Sanger Institute core teams, who contributed to the data generated in this sequencing project.

**Mapping/fingerprinting/archives:**

Sally Linsdell

Anth Cassidy

Rich Clark

Paul Hunt

Anne Angell

Deb Atkin

Carol Scott

Lucy Matthews

**Sequencing & sequence improvement:**

Andrew Dearlove

Karen Oliver

Rich Rance

Stuart McLaren

Deb Mason

Ann Joy

Chris Clee

Sophie Rockley

Joy Davies

Clare Murnane

Dan Hassan

Suze Clarke

Ruth Taylor

Ada Clarke

Michelle Smith

Phil Window

Tarryn Porter

Vicky Murray

Tracey Chillingworth

Doug Ormond

John Burton

Bill Bailey

Ali Hooper

Gary Hornett

Janet Squares

Linda Cornell

Paul Cummings

Ian Still

Paul Voak

Mel Robinson

Alan Wells

Tris Bellerby

Sarah Holmes

Sarah Sims

**Finishing:**

Siobhan Whitehead

Christine Lloyd

Paul Heath

Guy Griffiths

Jo Collins

Sarah Pelan

**Annotation:**

Toby Hunt

Matt Hardy

**Library construction:**

Matt Jones

Mike Quail

Dave Willey

Matthew Mayho

Jacqui Brown

Neil Marriott

**Data improvement:**

William Chow
